# Synthesis of six-membered silacycles by borane-catalyzed double sila-Friedel–Crafts reaction

**DOI:** 10.3762/bjoc.16.39

**Published:** 2020-03-17

**Authors:** Yafang Dong, Masahiko Sakai, Kazuto Fuji, Kohei Sekine, Yoichiro Kuninobu

**Affiliations:** 1 Interdisciplinary Graduate School of Engineering Sciences, Kyushu University, 6-1 Kasugakoen, Kasuga-shi, Fukuoka 816-8580, Japan; 2 Institute for Materials Chemistry and Engineering, Kyushu University, 6-1 Kasugakoen, Kasuga-shi, Fukuoka 816-8580, Japan

**Keywords:** borane, cyclic compound, organosilane, sila-Friedel–Crafts, silylation

## Abstract

We have developed a catalytic synthetic method to prepare phenoxasilins. A borane-catalyzed double sila-Friedel–Crafts reaction between amino group-containing diaryl ethers and dihydrosilanes can be used to prepare a variety of phenoxasilin derivatives in good to excellent yields. The optimized reaction conditions were also applicable for diaryl thioethers to afford their corresponding six-membered silacyclic products. The gram-scale synthesis of a representative bis(dimethylamino)phenoxasilin and the transformation of its amino groups have also been demonstrated.

## Introduction

**Scheme 1:**
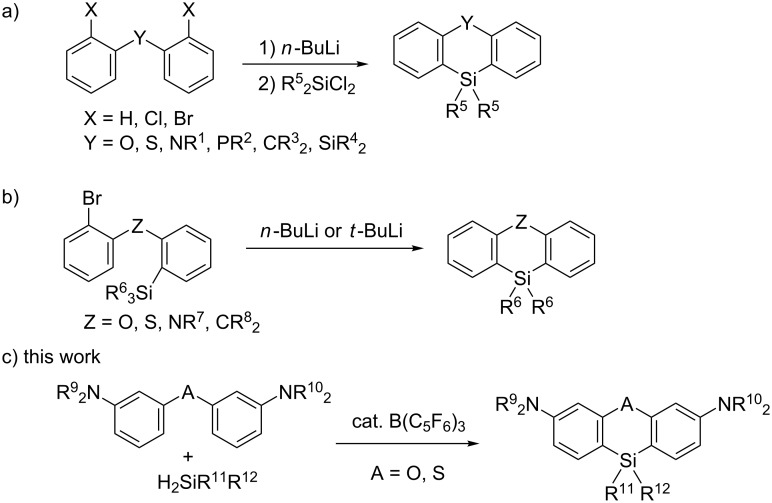
Synthetic methods of six-membered silacyclic compounds.

Six-membered silacyclic compounds, such as phenoxasilin and phenothiasilin derivatives, are attractive compounds for applications as organic electronic materials [1–4], ligands [5–10], and reagents [11–14]. Therefore, the development of new methods to construct silacyclic skeletons is highly desirable. These compounds are commonly synthesized upon the reaction of heteroatom-bridged dilithiated diaryl compounds, such as dilithiated diaryl ethers and dilithiated diaryl thioethers with a range of dichlorosilane derivatives (Scheme 1a) [15–24]. An intramolecular silylation via Si–C bond cleavage can also be used to prepare a variety of six-membered silacyclic derivatives (Scheme 1b) [25]. However, some problems still remain in terms of the functional group tolerance and versatility of these previously reported synthetic methods due to the use of a stoichiometric amount of the organolithium reagents. In addition, despite these contributions, catalytic reaction systems have not been developed as much [26–27].

The sila-Friedel–Crafts reaction is emerging as a powerful tool for C–H silylation [28–29]. In addition, intra- and intermolecular sila-Friedel–Crafts reactions have been recently developed [30–39], which have great potential as efficient synthetic strategies to construct silacycles. For example, the intramolecular C–H silylation of biphenylhydrosilanes can be used to prepare various silafluorene derivatives [30–34] and the ruthenium-catalyzed intermolecular Friedel–Crafts-type reaction of 2-phenylindole with a variety of dihydrosilanes affords indole-fused benzosiloles [39]. We have also contributed to the synthesis of silafluorenes from biphenyls and dihydrosilanes using a borane-catalyzed double sila-Friedel–Crafts reaction [40–41]. Subsequently, we envisaged that the catalytic reaction between diaryl ethers and dihydrosilanes may be a useful protocol to prepare phenoxasilin derivatives (Scheme 1c). Herein, we report a borane-catalyzed double sila-Friedel–Crafts reaction used for the synthesis of six-membered silacyclic compounds, such as phenoxasilin and phenothiasilin derivatives.

## Results and Discussion

A double sila-Friedel–Crafts reaction was initially investigated using diaryl ether **1a** and dihydrodiphenylsilane (**2a**) as model substrates (Table 1). Under the optimized reaction conditions used for the synthesis of silafluorenes in our previous report [40] (B(C_6_F_5_)_3_ (5.0 mol %) and 2,6-lutidine (7.5 mol %) in chlorobenzene at 100 °C), the desired reaction between **1a** with **2a** proceeded to give phenoxasilin **3a** in 60% yield (Table 1, entry 1). The structure of phenoxasilin **3a** was confirmed using single-crystal X-ray crystallography (see Supporting Information File 1 for details) [42]. Upon increasing the reaction temperature to 140 °C, the yield of **3a** was improved to 88% (Table 1, entry 2). Although the reaction in the presence of 3.0 mol % of the catalyst also proceeded efficiently (Table 1, entry 3, conditions A), the yield of **3a** decreased when compared to that obtained using 1.5 mol % of the catalyst (Table 1, entry 4). The best result was obtained in the absence of 2,6-lutidine by which phenoxasilin **3a** formed in 99% yield (Table 1, entry 5, conditions B).

**Table 1:** Optimization of the reaction conditions for the synthesis of phenoxalin **3a**.

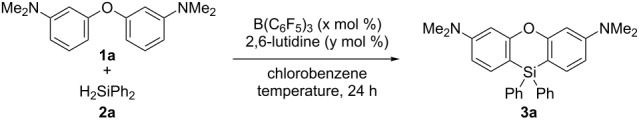				

entry^a^	x (mol%)	y (mol %)	temp (°C)	yield (%)

1	5.0	7.5	100	60
2	5.0	7.5	140	88
3	3.0	7.5	140	97
4	1.5	7.5	140	87
5	3.0	0	140	99

^a^**1a** (0.250 mmol), **2a** (0.750 mmol), chlorobenzene (0.4 mL).

**Scheme 2:**
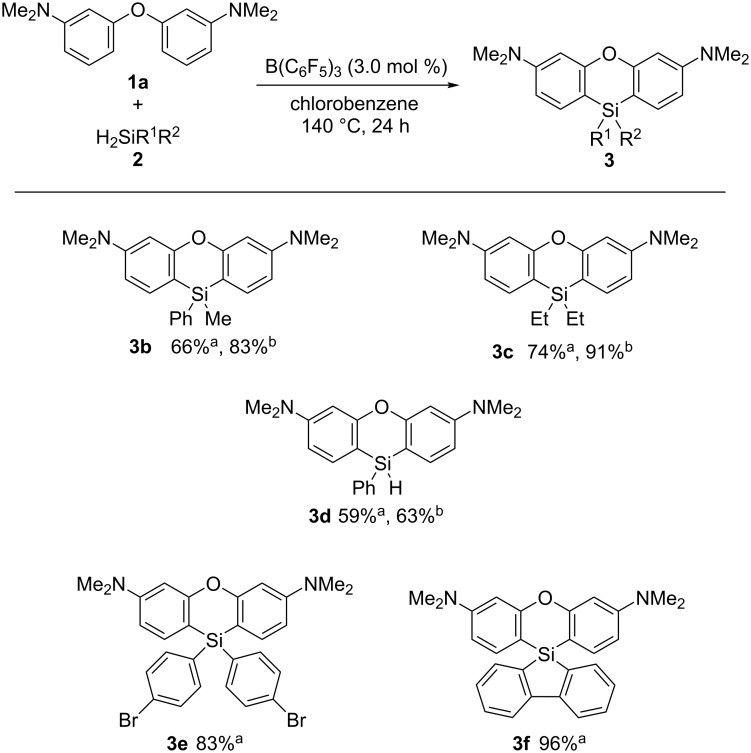
Scope of dihydrosilanes. Conditions: a: conditions B (Table 1, entry 5); b: conditions A (Table 1, entry 3).

Next, the scope of the dihydrosilane starting materials used in the reaction was investigated (Scheme 2). The reactions of phenylmethylsilane (**2b**) and diethyldihydrosilane (**2c**) afforded their corresponding phenoxasilin derivatives **3b** and **3c** in 66 and 74% yield, respectively. The yields of **3b** and **3c** were improved to 83 and 91% in the presence of a catalytic amount of 2,6-lutidine, probably due to the acceleration of the deprotonation step by 2,6-lutidine [33]. In the case of phenylsilane (**2d**), the phenoxasilin product **3d** was formed in 59% yield using conditions B and in 63% yield under conditions A. Di(4-bromophenyl)dihydrosilane (**2e**) was transformed successfully into phenoxasilin **3e** in 83% yield without loss of the bromine substituent. The reaction system was also applicable for 9,9-dihydro-5-silafluorene (**2f**), which gave the spiro-type phenoxasilin **3f** in 96% yield.

**Scheme 3:**
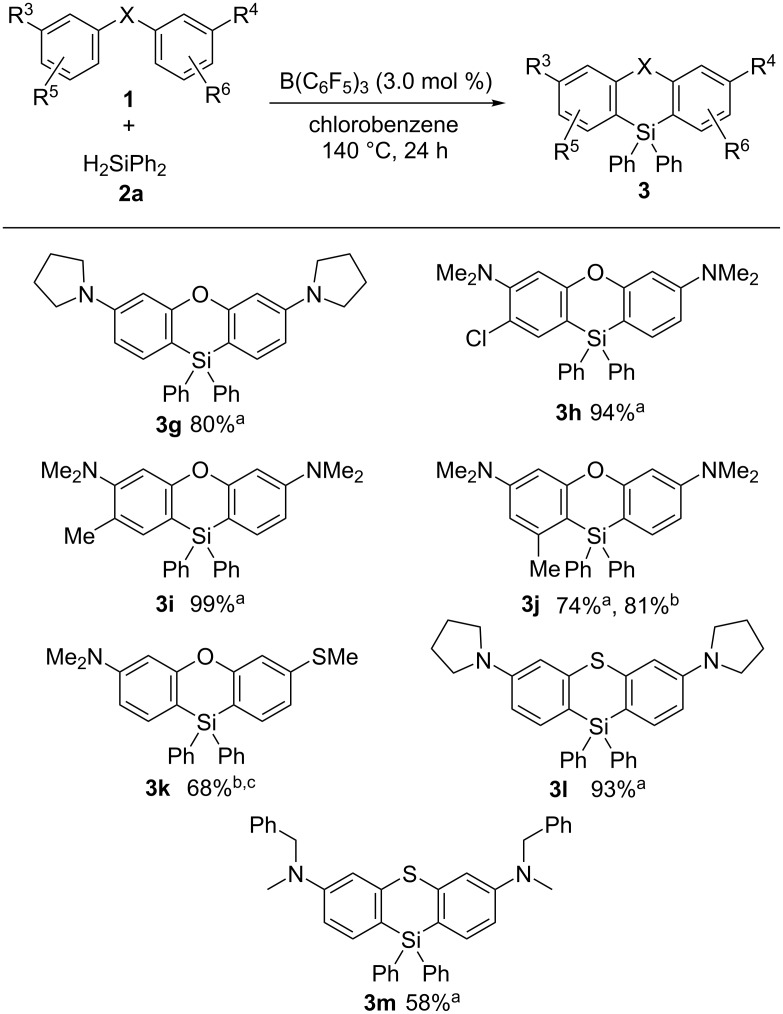
Scope of diaryl ether and diaryl thioether derivatives. Conditions: a: conditions B (Table 1, entry 5); b: conditions A (Table 1, entry 3). c: temperature 180 °C.

We then investigated the scope of the starting biaryl ethers used in the reaction as well as related derivatives thereof using dihydrodiphenylsilane (**2a**, Scheme 3). Pyrrolidine-substituted diaryl ether **1b** was transformed into phenoxasilin **3g** in 80% yield. Also, the chloro-substituted diaryl ether gave its corresponding phenoxasilin **3h** in 94% yield without affecting the chlorine substituent. The methyl-substituted phenoxasilin derivatives **3i** and **3j** were formed in good yield despite of the steric hindrance of the methyl group in **3j**. When one of the NMe_2_ groups was replaced with a SMe group, a mixture of the corresponding phenoxasilin product (**3k**) and the hydrosilane compound (**3k′**) was obtained via a single sila-Friedel–Crafts reaction in 35% yield in the presence of 2,6-lutidine (**3k**:**3k′** = 63:37). This result was probably due to the weaker electron-donating ability of the SMe group compared to that of NMe_2_. The double C–H silylation reaction proceeds efficiently upon increasing the temperature to 180 °C that afforded the mixture (**3k**:**3k′** = 92:8) in 68% yield. The reaction system can also be applied to the synthesis of phenothiasilin **3l** that was obtained in 93% yield starting from diaryl thioether **1g**. *N*-(Benzyl)methylamine-substituted diaryl thioether **1h** was also transformed into phenothiasilin **3m** in 58% yield. The corresponding six-membered silacycles were not formed using *N*-aryl-bridged biaryls as substrates.

**Scheme 4:**
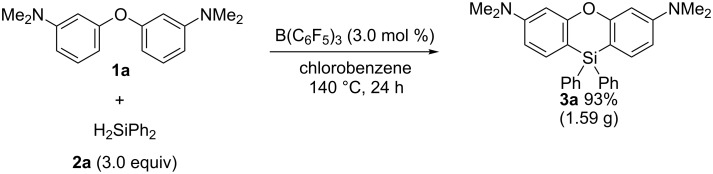
Gram-scale Synthesis of **3a**.

To test the applicability of the method, a gram-scale synthesis of phenoxasilin **3a** was carried out (Scheme 4). The reaction of diaryl ether **1a** (1.00 g) with dihydrodiphenylsilane (**2a**, 2.16 g) in the presence of a catalytic amount of B(C_6_F_5_)_3_ afforded phenoxasilin **3a** in 93% yield (1.59 g).

**Scheme 5:**
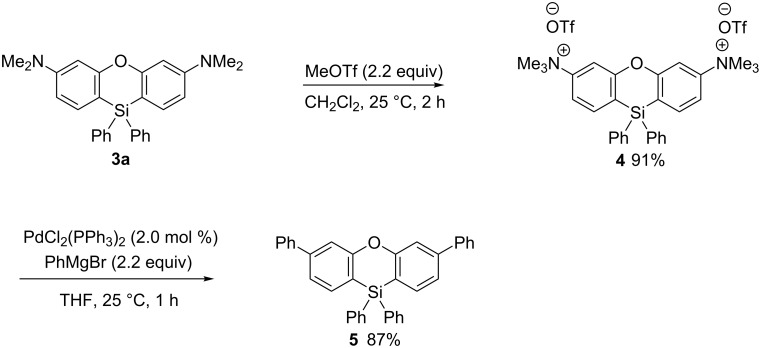
Transformation of the amino groups in **3a**.

Finally, the transformation of the amino groups in phenoxasilin **3a** into phenyl groups was carried out (Scheme 5). First, the ammonium salt **4** was prepared by treating **3a** with MeOTf followed by a palladium-catalyzed cross-coupling reaction with the Grignard reagent (PhMgBr) that afforded the desired diphenylated phenoxasilin **5** in 87% yield [43].

## Conclusion

In summary, we have developed a new catalytic synthetic method to prepare six-membered silacyclic compounds, such as phenoxasilin and phenothiasilin derivatives, using a double sila-Friedel–Crafts reaction. The reaction system is applicable to diaryl ethers with halogen substituents or sterical hindrance. A gram-scale synthesis of phenoxasilins and transformation of the amino groups in the phenoxasilin product were also achieved. We hope that the developed protocol will prove to be a useful and efficient method to synthesize six-membered silacyclic compounds.

## Supporting Information

File 1 Experimental procedures, compounds characterization data, and copies of ^1^H and ^13^C NMR spectra.

File 2 CIF file for **3a**.
