# Supplementary material for: Synthesis of six-membered silacycles by borane-catalyzed double sila-Friedel–Crafts reaction
Source: Beilstein J Org Chem. 2020 Mar 17;16:409–14. doi: 10.3762/bjoc.16.39 (PMC7113548; doi:10.3762/bjoc.16.39)
Supplement: File 1 — Experimental procedures, compounds characterization data, and copies of 1H and 13C NMR spectra. [file Beilstein_J_Org_Chem-16-409-s001.pdf]

BEILSTEIN JOURNAL OF ORGANIC CHEMISTRY

Supporting Information

for

Synthesis of six-membered silacycles by borane-catalyzed double sila-Friedel–Crafts reaction

Yafang Dong, Masahiko Sakai, Kazuto Fuji, Kohei Sekine and Yoichiro Kuninobu

Beilstein J. Org. Chem. **2020,** *16,* 409–414. doi:10.3762/bjoc.16.39

Experimental procedures, compounds characterization data, and copies of ^{1}H and ^{13}C NMR spectra

License and Terms: This is a supporting information file under the terms of the Creative Commons Attribution License (http://creativecommons.org/licenses/by/4.0). Please note that the reuse, redistribution and reproduction in particular requires that the authors and source are credited.
The license is subject to the *Beilstein Journal of Organic Chemistry* terms and conditions: (https://www.beilstein-journals.org/bjoc)

Contents

1. General S2
2. Substrate synthesis S2
3. General procedure for borane-catalyzed double sila-Friedel–Crafts reaction
and spectral data of six-membered silacycles **3** S5
4. X-ray structure of compound (**3a**) S10
5. NMR spectra S19
6. References..S42

1. General

All reactions were carried out using standard Schlenk techniques under an inert atmosphere. All reagents were purchased from commercial sources and used without further purification unless otherwise noted. NMR spectra were recorded on JEOL ECZ-400 (400 MHz for ^{1}H NMR, 100 MHz for ^{13}C NMR), JEOL ECA-600 (600 MHz for ^{1}H NMR, 150 MHz for ^{13}C NMR), JEOL JNM-LA400 (400 MHz for ^{1}H NMR, 100 MHz for ^{13}C NMR) spectrometers. Proton and carbon chemical shifts are reported relative to tetramethylsilane (TMS, δ 0.00 (^{1}H NMR, ^{13}C NMR)) or the residual solvent signal ($CHCl_3$ (δ 7.26 for ^{1}H NMR or δ 77.16 for ^{13}C NMR), CH_2Cl_2 (δ 5.23 for ^{1}H NMR or δ 53.84 for ^{13}C NMR)) was used as an internal reference. HRMS were measured on a JEOL JMS-700 spectrometer. Bis(4-bromophenyl)silane[1] and 5*H*-dibenzo[*b*,*d*]silole[2] were prepared according to the literature procedures or modified procedures.

2. Substrate synthesis

3-Bromo-*N*,*N*-dimethylaniline (S1)

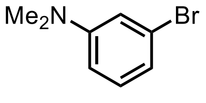

Compound **S1** was synthesized according to the reported method.[3] A mixture of 3-bromoaniline (10.0 mmol), iodomethane (22.0 mmol) and K_2CO_3 (22.0 mmol) in DMF (80 mL) was refluxed at 75 °C. After completion of the reaction as monitored by TLC, the mixture was poured into an aqueous $NaHCO_3$ solution and extracted with EtOAc. The organic layer was washed with brine, dried over Na_2SO_4 and concentrated in vacuo. Purification by column chromatography on silica gel afforded *N*,*N*-dimethylaniline (1.80 g, 90%). ^{1}H NMR (400 MHz, $CDCl_3$): *δ* 7.08 (dd, *J* = 8.2, 8.2 Hz, 1H), 6.84–6.81 (m, 2H), 6.64–6.61 (m, 1H), 2.94 (s, 6H). The analytical data is in accordance with the previous report.[4]

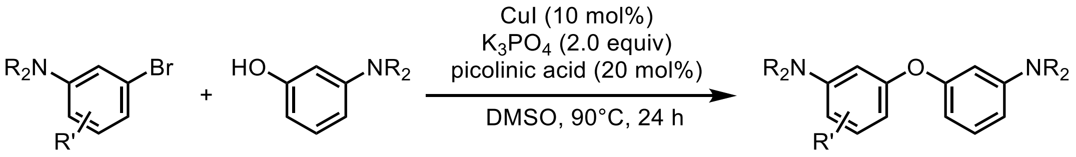

Scheme S1: Synthesis of diaryl ethers **1a**.

3,3'-Oxybis(*N*,*N*-dimethylaniline) (1a)

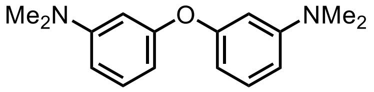

Compound **1a** was synthesized according to the reported method.[5] To a mixture of 3-(dimethylamino)phenol (987 mg, 7.20 mmol), CuI (114 mg, 0.600 mmol), 2-picolinic acid (148 mg, 1.20 mmol) and K_3PO_4 (2.55 g, 12.0 mmol) in DMSO (15 mL) at room temperature was added 3-bromo-*N*,*N*-dimethylaniline (**S1**, 1.20 g, 6.00 mmol) and the mixture was stirred vigorously at 90 °C for 24 h. The reaction mixture was cooled to room temperature, filtered with Celite and washed with EtOAc (150 mL). The filtrate was diluted with EtOAc (250 mL) and washed with brine (3 × 500 mL). The aqueous layers were extracted with EtOAc (2 ×

400 mL). The combined organic layers were dried over Na_2SO_4 and the solvent removed in vacuo. Purification by column chromatography (eluent: hexane/EtOAc 20:1 to 10:1) gave compound **1a** as white solid (1.14 g, 76% yield). ^{1}H NMR (400 MHz, $CDCl_3$) δ 7.15 (dd, *J* = 8.0, 8.0 Hz, 2H), 6.44–6.48 (m, 4H), 6.35 (dd, *J* = 8.0, 1.6 Hz, 2H), 2.93 (s, 12H); ^{13}C NMR (100 MHz, $CDCl_3$) δ 158.5, 152.2, 129.9, 107.5, 106.9, 103.5, 40.7; HRMS(EI$^+$) Calcd for $C_{16}H_{20}N_2O$ ([M]$^+$) 256.1570, Found 256.1578.

1,1'-[Oxybis(3,1-phenylene)]dipyrrolidine (1b)

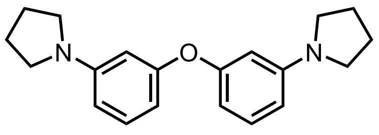

Compound **1b** was obtained in 56% yield (355 mg, 1.12 mmol) following the same method as described for **1a** by the reaction between 1-(3-bromophenyl)pyrrolidine[6] (452 mg, 2.00 mmol) and 3-(pyrrolidin-1-yl)phenol (392 mg, 2.40 mmol). ^{1}H NMR (400 MHz, $CDCl_3$) δ 7.13 (dd, *J* = 8.2, 8.2 Hz, 2H), 6.28–6.31 (m, 6H), 3.23–3.27 (m, 8H), 1.96–2.00 (m, 8H); ^{13}C NMR (100 MHz, $CDCl_3$) δ 158.5, 149.4, 129.9, 106.7, 105.8, 102.5, 47.8, 25.6; HRMS(EI$^+$) Calcd for $C_{20}H_{24}N_2O$ ([M]$^+$) 308.1883, Found 308.1890.

5-Bromo-2-chloro-*N*,*N*-dimethylaniline (S2)[7]

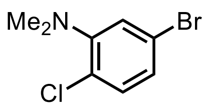

To a solution of 5-bromo-2-chloroaniline (1.65 g, 8.00 mmol, 1.0 equiv) in dry DMF (30 mL) were added MeI (2.49 mL, 40.0 mmol, 5.0 equiv) and NaH (1.75 g, 40.0 mmol, 5.0 equiv; 60 wt % in mineral oil). After 1 h, the reaction was quenched with water (15 mL) and brine (25 mL) and Et_2O (25 mL) were added. The organic layer was separated, washed with brine (2 × 25 mL), dried over anhydrous $MgSO_4$, filtered and concentrated. The crude product was then purified by column chromatography (eluent: hexane/EtOAc 19:1) on silica gel to give 5-bromo-2-chloro-*N*,*N*-dimethylaniline as colorless oil (1.66 g, 89%). ^{1}H NMR (400 MHz, $CDCl_3$) δ 7.19 (d, *J* = 8.2 Hz, 1H), 7.15 (d, *J* = 2.3 Hz, 1H), 7.05 (dd, *J* = 8.2, 2.3 Hz, 1H), 2.81 (s, 6H); ^{13}C NMR (100 MHz, $CDCl_3$) δ 151.7, 131.9, 127.1, 126.0, 123.4, 120.9, 43.7; HRMS(EI$^+$) Calcd for C_8H_9BrClN ([M]$^+$) 232.9607, Found 232.9609.

2-Chloro-5-(3-(dimethylamino)phenoxy)-*N*,*N*-dimethylaniline (1c)

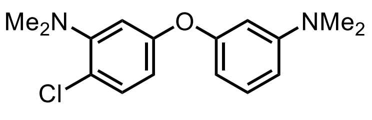

Compound **1c** was obtained in 62% yield (449 mg, 1.55 mmol) following the same method as described for **1a** by the reaction between **S2** (586 mg, 2.50 mmol) and 3-(dimethylamino)phenol (412 mg, 3.00 mmol). ^{1}H NMR (400 MHz, $CDCl_3$) δ 7.24 (d, *J* = 8.7 Hz, 1H), 7.17 (dd, *J* = 8.2, 8.2 Hz, 1H), 6.78 (d, *J* = 2.7 Hz, 1H), 6.56 (dd, *J* = 8.7, 2.7 Hz, 1H), 6.49 (dd, *J* = 8.5, 2.5 Hz, 1H), 6.39 (dd, *J* = 2.3, 2.3 Hz, 1H), 6.32 (dd, *J* = 8.0, 2.1 Hz, 1H), 2.93 (s, 6H), 2.79 (s, 6H); ^{13}C NMR (100 MHz, $CDCl_3$) δ 157.9, 156.9, 152.2, 151.6, 131.2, 130.1, 121.8, 112.9, 111.0, 108.0, 106.8, 103.4, 43.8, 40.6; HRMS(EI$^+$) Calcd for $C_{16}H_{19}ClN_2O$ ([M]$^+$) 290.1180, Found 290.1187.

5-(3-(Dimethylamino)phenoxy)-*N*,*N*,2-trimethylaniline (1d)

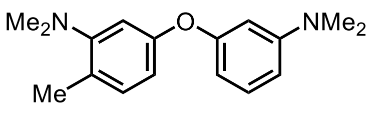

Compound **1d** was obtained in 52% yield (354 mg, 1.31 mmol) following the same method as described for **1a** by the reaction between 3-bromo-*N*,*N*,6-trimethylaniline[7] (535 mg, 2.50 mmol) and 3-(dimethylamino)phenol (412 mg, 3.00 mmol). ^{1}H NMR (400 MHz, $CDCl_3$) δ 7.15 (dd, J = 8.2, 8.2 Hz, 1H), 7.07 (d, J = 8.2 Hz, 1H), 6.76 (d, J = 2.7 Hz, 1H), 6.58 (dd, J = 8.2, 2.7 Hz, 1H), 6.46 (dd, J = 8.0, 2.1 Hz, 1H), 6.41 (dd, J = 2.3, 2.3 Hz, 1H), 6.31 (dd, J = 7.8, 1.8 Hz, 1H), 2.93 (s, 6H), 2.67 (s, 6H), 2.28 (s, 3H); ^{13}C NMR (100 MHz, $CDCl_3$) δ 158.7, 155.8, 154.0, 152.2, 131.8, 130.0, 126.6, 112.7, 109.9, 107.4, 106.4, 103.1, 44.2, 40.7, 18.1; HRMS(EI+) Calcd for $C_{17}H_{22}N_2O$ ($[M]^+$) 270.1727, Found 270.1732.

3-Bromo-*N*,*N*,5-trimethylaniline (S3)[7]

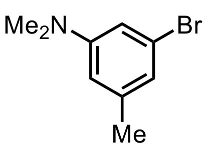

The same method as described for **S2**. 3-Bromo-5-methylaniline (1.12 g, 6.00 mmol, 1.0 equiv), K_2CO_3 (7.50 g, 54.0 mmol, 9.0 equiv), MeI (4.26 g, 30.0 mmol, 5.0 equiv) and DMF (15 mL). The crude product was then purified by column chromatography (eluent: hexane/EtOAc 9:1) on silica gel to give the desired compound as colorless oil (1.00 g, 78%). ^{1}H NMR (400 MHz, $CDCl_3$) δ 6.67–6.65 (m, 2H), 6.43 (s, 1H), 2.92 (s, 6H), 2.27 (s, 3H); ^{13}C NMR (100 MHz, $CDCl_3$) δ 151.7, 140.5, 123.3, 120.2, 112.6, 111.9, 40.6, 21.8; HRMS(EI^+) Calcd for $C_9H_{12}BrN$ ($[M]^+$) 213.0148, Found 213.0154.

3-(3-(Dimethylamino)phenoxy)-*N*,*N*,5-trimethylaniline (1e)

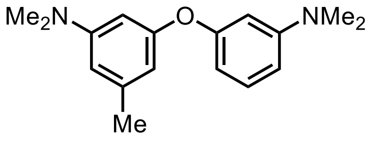

Compound **1e** was obtained in 54% yield (369 mg, 1.36 mmol) following the same method as described for **1a** by the reaction between **S3** (535 mg, 2.50 mmol) and 3-(dimethylamino)phenol (412 mg, 3.00 mmol). ^{1}H NMR (400 MHz, $CDCl_3$) δ 7.15 (dd, J = 8.0, 8.0 Hz, 1H), 6.44–6.48 (m, 2H), 6.35 (dd, J = 8.0, 1.1 Hz, 1H), 6.27 (dd, J = 2.3, 2.3 Hz, 2H), 6.19 (s, 1H), 2.93 (s, 6H), 2.91 (s, 6H), 2.26 (s, 3H); ^{13}C NMR (100 MHz, $CDCl_3$) δ 158.4, 158.4, 152.1, 152.0, 140.0, 129.9, 108.3, 107.8, 107.5, 107.0, 103.6, 100.8, 40.8, 40.7, 22.1; HRMS(EI^+) Calcd for $C_{17}H_{22}N_2O$ ($[M]^+$) 270.1727, Found 270.1729.

***N*,*N*-Dimethyl-3-(3-(methylthio)phenoxy)aniline (1f)**

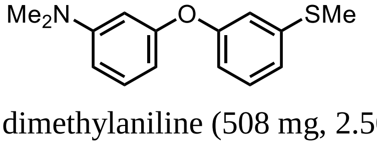

Compound **1f** was obtained in 44% yield (285 mg, 1.10 mmol) following the same method as described for **1a** by the reaction between 3-bromo-*N*,*N*-dimethylaniline (508 mg, 2.50 mmol) and 3-(methylthio)phenol (412 mg, 3.00 mmol). ^{1}H NMR (400 MHz, $CDCl_3$) δ 7.15–7.23 (m, 2H), 6.91–6.96 (m, 2H), 6.76 (dd, J = 8.2, 1.1 Hz, 1H), 6.49 (dd, J = 8.0, 2.1 Hz, 1H), 6.41 (dd, J = 2.3, 2.3 Hz, 1H), 6.33 (dd, J = 8.2, 1.8 Hz, 1H), 2.93 (s, 6H), 2.45 (s, 3H); ^{13}C NMR (100 MHz, $CDCl_3$) δ 158.2, 157.8, 152.2, 140.2, 130.1, 129.9, 120.8, 116.4, 115.2, 108.0, 107.1, 103.7, 40.6, 15.7; HRMS(EI^+) Calcd for $C_{15}H_{17}NOS$ ($[M]^+$) 259.1025, Found 259.1032.

Bis(3-(pyrrolidin-1-yl)phenyl)sulfane (1g)

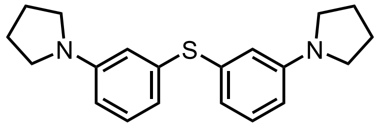

Compound **1g** was synthesized according to a reported method.[8] A Schlenk flask was charged with bis(3-bromophenyl)sulfane[9] (313 mg, 0.910 mmol, 1.00 equiv), pyrrolidine (226 μL, 2.73 mmol, 3.0 equiv), NaO*t*-Bu (262 mg, 2.73 mmol, 3.0 equiv), Pd(dba)$_2$ (21.6 mg, 80.0 μmol, 4.00 mol %), BINAP (67.0 mg, 110 μmol, 12.0 mol %), and toluene (1.0 mL) under N_2. The flask was immersed in an oil bath and heated to 80 °C with stirring overnight. The mixture was cooled to room temperature, filtered over Celite, and concentrated. The crude product was then purified by column chromatography (eluent: hexane) on silica gel to give **1g** as white solid (173 mg, 59%). ^{1}H NMR (400 MHz, CDCl$_3$) δ 7.12 (dd, *J* = 7.8, 7.8 Hz, 2H), 6.62–6.64 (m, 4H), 6.42–6.44 (m, 2H), 3.22–3.25 (m, 8H), 1.96–1.99 (m, 8H); ^{13}C NMR (100 MHz, CDCl$_3$) δ 148.4, 136.6, 129.7, 118.0, 114.0, 110.4, 47.7, 25.6; HRMS(EI$^+$) Calcd for $C_{20}H_{24}N_2S$ ([M]$^+$) 324.1655, Found 324.1660.

3,3'-Thiobis(*N*-benzyl-*N*-methylaniline) (1h)

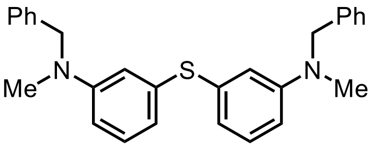

Compound **1h** was obtained in 71% yield following the same method as described for **1g** by the reaction between bis(3-bromophenyl)sulfan[9] (258 mg, 0.750 mmol) and benzylmethylamine (218 mg, 1.80 mmol). ^{1}H NMR (400 MHz, CDCl$_3$) δ 7.29 (dd, *J* = 7.3, 7.3 Hz, 4H), 7.16–7.25 (m, 6H), 7.10 (t, *J* = 8.0 Hz, 2H), 6.76 (dd, *J* = 2.1, 2.1 Hz, 2H), 6.66 (d, *J* = 7.8 Hz, 2H), 6.59 (dd, *J* = 8.5, 2.5 Hz, 2H), 4.48 (s, 4H), 2.97 (s, 6H); ^{13}C NMR (100 MHz, CDCl$_3$) δ 150.3, 138.8, 136.5, 129.8, 128.7, 127.0, 126.8, 119.1, 114.5, 111.1, 56.6, 38.7; HRMS(EI$^+$) Calcd for $C_{28}H_{28}N_2S$ ([M]$^+$) 424.1968, Found 424.1971.

3. General procedure for the borane-catalyzed double sila-Friedel–Crafts reaction and spectral data of six-membered silacycles 3

A test tube with a screw cap equipped with a magnetic stirring bar was charged diaryl ether **1a** (64.1 mg, 0.250 mmol, 1.00 equiv) and tris(pentafluorophenyl)borane ($B(C_6F_5)_3$, 3.80 mg, 0.00750 mmol, 3.0 mol %). The tube was evacuated and filled with nitrogen. Chlorobenzene (0.40 mL) was added via syringe. Diphenylsilane **2a** (0.140 mL, 0.750 mmol, 3.0 equiv) was then added to the mixture (if necessary, 2,6-lutidine (2.20 μL, 0.0190 mmol, 7.5 mol %) was also added). The test tube was closed with a screw cap and the reaction mixture was stirred at 140 °C (oil bath) for 24 h. After completion of the reaction, the mixture was cooled to room temperature. Dichloromethane (10 mL) were added. The crude product was purified by column chromatography on silica gel (eluent: hexane/EtOAc 25:1) to give compound **3a** as white solid (106 mg, 97% yield).

Phenoxasilin 3a

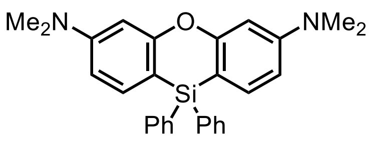

^{1}H NMR (400 MHz, $CDCl_3$) δ 7.58 (dd, J = 7.8, 1.4 Hz, 4H), 7.28–7.38 (m, 8H), 6.52–6.54 (m, 4H), 2.98 (s, 12H); ^{13}C NMR (100 MHz, $CDCl_3$) δ 162.1, 153.2, 136.09, 136.06, 135.9, 129.4, 127.9, 108.3, 102.3, 100.6, 40.3; HRMS(EI^+) Calcd for $C_{28}H_{28}N_2OSi$ ($[M]^+$) 436.1965, Found 436.1972.

Phenoxasilin 3b

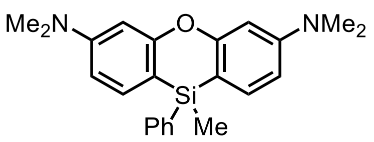

Compound **3b** was obtained as white solid (62.5 mg, 83%) from diaryl ether **1a** (51.3 mg, 0.200 mmol) with 2,6-lutidine. ^{1}H NMR (400 MHz, $CDCl_3$) δ 7.52–7.54 (m, 2H), 7.29–7.31 (m, 5H), 6.54 (d, J = 2.5 Hz, 1H), 6.50–6.52 (m, 3H), 2.99 (s, 12H), 0.70 (s, 3H); ^{13}C NMR (100 MHz, $CDCl_3$) δ 161.7, 153.1, 138.6, 135.3, 134.8, 129.2, 127.9, 108.2, 104.0, 100.6, 40.4, -2.0; HRMS(EI^+) Calcd for $C_{23}H_{26}N_2OSi$ ($[M]^+$) 374.1809, Found 374.1811.

Phenoxasilin 3c

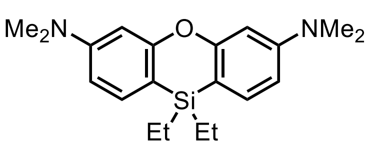

Compound **3c** was obtained as white solid (77.2 mg, 91%) from diaryl ether **1a** (64.1 mg, 0.250 mmol) with 2,6-lutidine. ^{1}H NMR (400 MHz, $CDCl_3$) δ 7.32 (d, J = 8.2 Hz, 2H), 6.54 (dd, J = 8.0, 2.1 Hz, 2H), 6.47 (d, J = 2.3 Hz, 2H), 2.99 (s, 12H), 0.85–0.90 (m, 10H); ^{13}C NMR (100 MHz, $CDCl_3$) δ 162.0, 152.8, 134.8, 107.8, 103.5, 100.7, 40.4, 7.8, 6.6; HRMS(EI^+) Calcd for $C_{20}H_{28}N_2OSi$ ($[M]^+$) 340.1965, Found 340.1974.

Phenoxasilin 3d

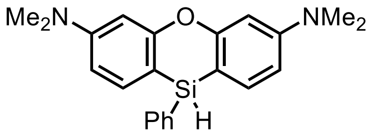

Compound **3d** was obtained as white solid (56.6 mg, 63%) from diaryl ether **1a** (64.8 mg, 0.253 mmol) with 2,6-lutidine. ^{1}H NMR (400 MHz, $CDCl_3$) δ 7.60 (dd, J = 7.5, 1.6 Hz, 2H), 7.28–7.38 (m, 5H), 6.52 (dd, J = 6.4, 2.3 Hz, 4H), 5.42 (s, 1H), 3.00 (s, 12H); ^{13}C NMR (100 MHz, $CDCl_3$) δ 161.9, 153.3, 136.3, 136.0, 135.5, 129.8, 128.1, 108.2, 100.6, 100.2, 40.3; HRMS(EI^+) Calcd for $C_{22}H_{24}N_2OSi$ ($[M]^+$) 360.1652, Found 360.1656.

Phenoxasilin 3e

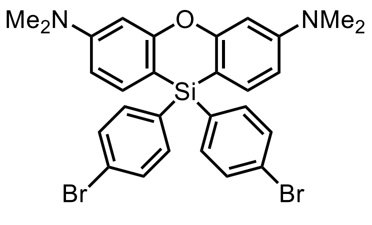

Compound **3e** was obtained as white solid (98.1 mg, 83%) from diaryl ether **1a** (51.3 mg, 0.200 mmol). ^{1}H NMR (400 MHz, $CDCl_3$) δ 7.40–7.47 (m, 8H), 7.30 (dd, J = 4.1 Hz, 2H), 6.51–6.55 (m, 4H), 3.00 (s, 12H); ^{13}C NMR (100 MHz, $CDCl_3$) δ 162.1, 153.3, 137.4, 135.7, 134.6, 131.2, 124.7, 108.4, 100.9, 100.6, 40.3; HRMS(EI^+) Calcd for $C_{28}H_{26}Br_2N_2OSi$ ($[M]^+$) 592.0176, Found 592.1083.

Phenoxasilin 3f

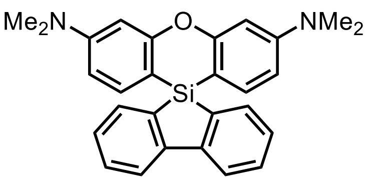

Compound **3f** was obtained as white solid (83.9 mg, 96%) from diaryl ether **1a** (51.3 mg, 0.200 mmol). ^{1}H NMR (400 MHz, $CDCl_3$) δ 7.89 (d, J = 7.3 Hz, 2H), 7.43–7.47 (m, 4H), 7.19 (dd, J = 7.3, 7.3 Hz, 2H), 6.99 (d, J = 8.2 Hz, 2H), 6.58 (d, J = 2.3 Hz, 2H), 6.39 (dd, J = 8.2, 2.3 Hz, 2H), 2.99 (s, 12H); ^{13}C NMR (100 MHz, $CDCl_3$) δ 163.2, 153.6, 148.9, 137.5, 135.9, 134.5, 130.9, 127.9, 120.7, 108.1, 100.6, 99.4, 40.3; HRMS(EI^{+}) Calcd for $C_{28}H_{26}N_2OSi$ ([M]$^{+}$) 434.1809, Found 434.1814.

Phenoxasilin 3g

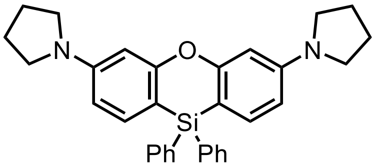

Compound **3g** was obtained as white solid (78.1 mg, 80%) from diaryl ether **1b** (61.7 mg, 0.200 mmol). ^{1}H NMR (400 MHz, CD_2Cl_2) δ 7.55 (dd, J = 8.0, 1.6 Hz, 4H), 7.30–7.39 (m, 8H), 6.40 (dd, J = 8.2, 2.3 Hz, 2H), 6.34 (d, J = 2.3 Hz, 2H), 3.30–3.33 (m, 8H), 1.99–2.02 (m, 8H); ^{13}C NMR (100 MHz, CD_2Cl_2) δ 162.2, 151.0, 136.7, 136.1, 135.9, 129.6, 128.0, 108.4, 101.1, 99.9, 47.9, 25.8; HRMS(EI^{+}) Calcd for $C_{32}H_{32}N_2OSi$ ([M]$^{+}$) 488.2278, Found 488.2284.

Phenoxasilin 3h

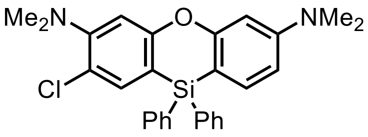

Compound **3h** was obtained as white solid (92.6 mg, 94%) from diaryl ether **1c** (61.1 mg, 0.210 mmol). ^{1}H NMR (400 MHz, $CDCl_3$) δ 7.57 (d, J = 6.4 Hz, 4H), 7.33–7.44 (m, 8H), 6.89 (s, 1H), 6.52–6.57 (m, 2H), 3.01 (s, 6H), 2.86 (s, 6H); ^{13}C NMR (100 MHz, $CDCl_3$) δ 161.8, 160.0, 153.3, 153.1, 136.3, 136.1, 136.0, 134.9, 129.9, 128.1, 122.0, 111.0, 109.7, 108.7, 101.2, 100.4, 43.7, 40.3; HRMS(EI^{+}) Calcd for $C_{28}H_{27}ClN_2OSi$ ([M]$^{+}$) 470.1576, Found 470.1582.

Phenoxasilin 3i

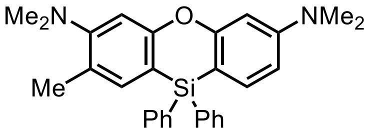

Compound **3i** was obtained as white solid (95.2 mg, >99%) from diaryl ether **1d** (54.1 mg, 0.200 mmol). ^{1}H NMR (400 MHz, CD_2Cl_2) δ 7.56 (dd, J = 8.0, 1.6 Hz, 4H), 7.32–7.39 (m, 7H), 7.26 (s, 1H), 6.82 (s, 1H), 6.56 (dd, J = 8.2, 2.3 Hz, 1H), 6.50 (d, J = 2.3 Hz, 1H), 2.99 (s, 6H), 2.74 (s, 6H), 2.24 (s, 3H); ^{13}C NMR (100 MHz, CD_2Cl_2) δ 162.2, 160.0, 156.4, 153.7, 137.2, 136.1, 136.0, 135.9, 129.9, 128.2, 126.4, 108.7, 108.6, 107.6, 101.6, 100.5, 43.9, 40.3, 18.3; HRMS(EI^{+}) Calcd for $C_{29}H_{30}N_2OSi$ ([M] $^{+}$) 450.2122, Found 450.2127.

Phenoxasilin 3j

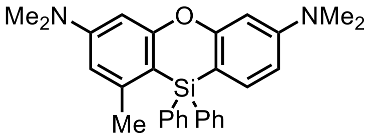

Compound **3j** was obtained as white solid (72.5 mg, 81%) from diaryl ether **1e** (54.1 mg, 0.200 mmol) with 2,6-lutidine. ^{1}H NMR (400 MHz, $CDCl_3$) δ 7.63 (dd, J = 7.8, 1.8 Hz, 4H), 7.30–7.35 (m, 6H), 7.24 (t, J = 4.3 Hz, 1H), 6.43-6.48 (m, 3H), 6.36 (d, J = 2.3 Hz, 1H), 3.00 (s, 6H), 2.96 (s, 6H), 2.17 (s, 3H); ^{13}C NMR (100 MHz, $CDCl_3$) δ 162.7, 161.4, 153.1, 153.0, 146.1, 136.3, 136.1, 136.0, 129.3, 127.9, 109.4, 108.3, 103.6, 101.5, 100.0, 98.8, 40.3, 40.2, 24.7; HRMS(EI^{+}) Calcd for $C_{29}H_{30}N_2OSi$ ([M]$^{+}$) 450.2122, Found 450.2126.

Phenoxasilin 3k

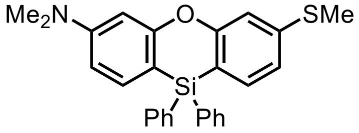

The mixture of **3k** and the hydrosilane **3k'** produced via a single sila-Friedel–Crafts reaction was obtained as white solid (60.1 mg, 68% yield of **3k** and **3k'**(**3k**:**3k'** = 92:8)) from diaryl ether **1f** (52.0 mg, 0.200 mmol). ^{1}H NMR (400 MHz, $CDCl_3$) δ 7.64 (dd, J = 7.8, 1.4 Hz, 4H), 7.38–7.50 (m, 8H), 7.14 (d, J = 1.1 Hz, 1H), 7.04 (dd, J = 8.0, 1.6 Hz, 1H), 6.62 (td, J = 8.9, 2.3 Hz, 2H), 3.08 (s, 6H), 2.58 (s, 3H); ^{13}C NMR (100 MHz, $CDCl_3$) δ 161.8, 161.0, 153.3, 142.7, 136.0, 135.9, 135.5, 135.1, 129.8, 128.0, 120.6, 114.6, 112.6, 108.6, 101.5, 100.6, 40.3, 15.1; HRMS(EI^{+}) Calcd for $C_{27}H_{25}NOSSi$ ([M]$^{+}$) 439.1421, Found 439.1427.

Phenothiasiline 3l

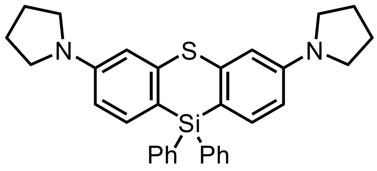

Compound **3l** was obtained as white solid (46.9 mg, 93%) from diaryl ether **1g** (32.6 mg, 0.100 mmol). ^{1}H NMR (400 MHz, CD_2Cl_2) δ 7.46 (dd, J = 8.0, 1.6 Hz, 4H), 7.31–7.41 (m, 6H), 7.21 (d, J = 8.2 Hz, 2H), 6.67 (d, J = 2.3 Hz, 2H), 6.44 (dd, J = 8.2, 2.3 Hz, 2H), 3.27–3.30 (m, 8H), 1.97–2.00 (m, 8H); ^{13}C NMR (100 MHz, CD_2Cl_2) δ 149.0, 144.2, 136.8, 136.3, 135.2, 129.7, 128.1, 115.7, 110.2, 109.8, 47.7, 25.7; HRMS(EI^{+}) Calcd for $C_{32}H_{32}N_2SSi$ ([M]$^{+}$) 504.2050, Found 504.2057.

Phenothiasiline 3m

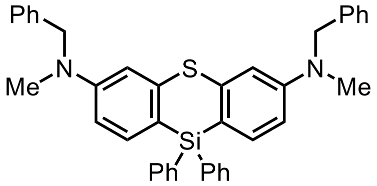

Compound **3m** was obtained as white solid (70.4 mg, 58%) from diaryl ether **1h** (85.5 mg, 0.200 mmol). ^{1}H NMR (400 MHz, $CDCl_3$) δ 7.50 (dd, J = 8.0, 1.6 Hz, 4H), 7.29–7.40 (m, 11H), 7.18–7.25 (m, 7H), 6.88 (d, J = 2.3 Hz, 2H), 6.59 (dd, J = 8.2, 2.3 Hz, 2H), 4.54 (s, 4H), 3.03 (s, 6H); ^{13}C NMR (100 MHz, $CDCl_3$) δ 150.5, 144.3, 138.6, 136.8, 136.3, 134.4, 129.6, 128.8, 127.9, 127.1, 126.7, 117.6, 110.3, 56.1, 38.5 (one carbon is missing); HRMS(EI^{+}) Calcd for $C_{40}H_{36}N_2SSi$ ([M]$^{+}$) 604.2363, Found 604.2370.

Ammonium Salt 4

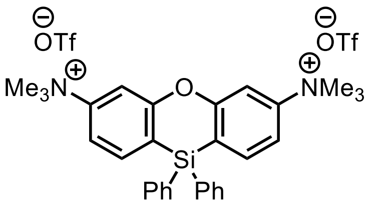

A dry round-bottomed flask equipped with a magnetic stirring bar was charged with **3a** (175 mg, 0.400 mmol, 1.00 equiv) and CH_2Cl_2 (5 mL). To the resultant stirring solution was added dropwise MeOTf (144 mg, 0.880 mmol, 2.20 equiv) at room temperature. The solution was stirred at room temperature for 2 h. The reaction mixture was concentrated to remove CH_2Cl_2 and the residue was treated with Et_2O (20 mL). The resultant solid was filtered, washed with Et_2O and hexane, and dried under vacuum to give **4** as white solid (279 mg, 91%). ^{1}H NMR (400 MHz, DMSO-d_6) δ 7.96 (d, J = 8.2 Hz, 2H), 7.84–7.90 (m, 4H), 7.40–7.57 (m, 12H), 3.66 (s, 18H); ^{13}C NMR (150 MHz, DMSO-d_6) δ 159.3, 150.2, 137.5, 135.4, 131.3, 130.9, 128.6, 120.7 (q, J = 322 Hz), 117.6, 116.0, 110.8, 56.3; HRMS(FAB$^+$) Calcd for $C_{31}H_{34}F_3N_2O_4SSi$ ([M-$^-$OTf]$^+$) 615.1961, Found 615.1962.

Phenoxasilin 5

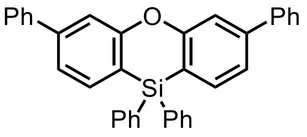

Compound **5** was synthesized according to a reported method. To a dry Schlenk flask equipped with a magnetic stirring bar was added compound **4** (153 mg, 0.200 mmol, 1.0 equiv) and $PdCl_2(PPh_3)_2$ (2.8 mg, 0.0040 mmol, 2.0 mol %). The flask was sealed with a rubber septum, and evacuated/filled with nitrogen. THF (1.5 mL) was added via syringe, and the resultant slurry was stirred for 5 min. Then phenylmagnesium bromide (0.5 M solution in THF, 0.88 mL, 0.44 mmol, 2.2 equiv) was added dropwise at room temperature. After 1 h, the reaction mixture was quenched with water (1.0 mL) and 6 M HCl (3 mL), and extracted with Et_2O. The organic extract was dried over Na_2SO_4, filtered, and concentrated. The crude product was purified by chromatography on silica gel (eluent: hexane/EtOAc 50:1) to give compound **5** as white solid (87.2 mg, 87% yield). ^{1}H NMR (400 MHz, $CDCl_3$) δ 7.63-7.68 (m, 10H), 7.53 (d, J = 1.4 Hz, 2H), 7.37–7.49 (m, 14H); ^{13}C NMR (100 MHz, $CDCl_3$) δ 160.9, 144.9, 140.5, 136.1, 136.0, 134.1, 130.2, 129.0, 128.3, 128.0, 127.4, 121.9, 116.8, 114.7; HRMS(EI$^+$) Calcd for $C_{36}H_{26}OSi$ ([M]$^+$) 502.1747, Found 502.1755.

4. X-ray structure of compound 3a

A suitable crystal of $C_{28}H_{28}N_2Si$, phenoxasilin **3a** was selected, and its X-ray diffraction data were collected on a Rigaku Saturn70 CCD area detector with graphite monochromated MoKα radiation (λ = 0.71070 Å). The crystal was kept at 123 K during data collection. The data were collected using ω scan in the θ range of 3.154 ≤ 2θ ≤ 62.02 deg. The data were corrected for Lorentz and polarization effects. The structures were solved by direct methods,[10] and expanded using Fourier techniques.[11] Hydrogen atoms were refined using the riding model. The final cycle of full-matrix least-squares refinement on F^2 was based on 14757 observed reflections and 585 variable parameters. Neutral atom scattering factors were taken from Cromer and Waber.[12] All calculations were performed using the Olex-2 crystallographic software package except for refinement,[13] which was performed using SHELXL-97.5 Details[14] of final refinement as well as the bond lengths and angles are summarized in the following Tables together with the numbering scheme employed, which were drawn with ORTEP at 50% probability ellipsoid.

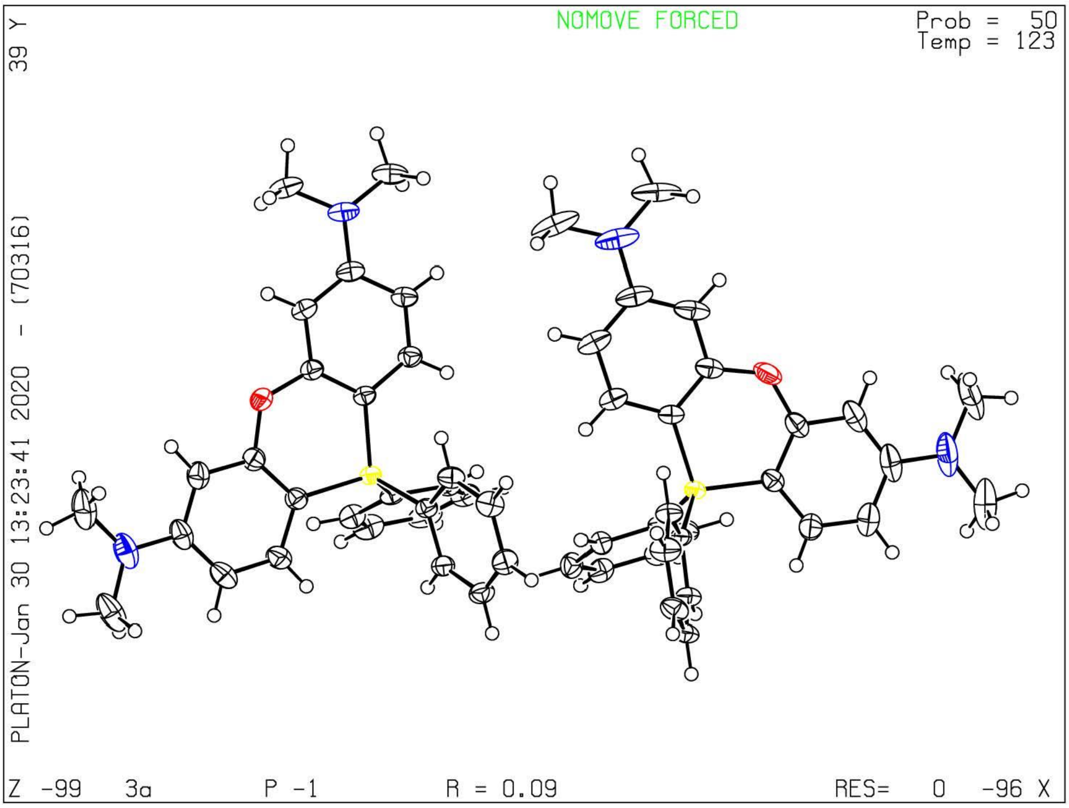

Table S1 Crystal data and structure refinement for 3a.

Identification code	3a
Empirical formula	$C_{28}H_{28}N_2OSi$
Formula weight	436.61
Temperature/K	123

Crystal system	triclinic
Space group	P-1
a/Å	12.4645(5)
b/Å	12.9987(5)
c/Å	16.7305(5)
α /°	83.723(3)
β /°	73.112(3)
γ /°	86.634(3)
Volume/Å^3	2577.23(17)
Z	5
ρ_{calc}g/cm^3	1.407
μ /mm^{-1}	0.140
F(000)	1160.0
Crystal size/mm^3	0.4 × 0.25 × 0.24
Radiation	MoKα (λ = 0.71073)
2Θ range for data collection/°	3.154 to 62.02
Index ranges	-17 ≤ h ≤ 17, -18 ≤ k ≤ 18, -24 ≤ l ≤ 23
Reflections collected	45536
Independent reflections	14757 [R_{int} = 0.0927, R_{sigma} = 0.0651]
Data/restraints/parameters	14757/0/585
Goodness-of-fit on F^2	1.054
Final R indexes [I>=2σ (I)]	R_1 = 0.0867, wR_2 = 0.2276
Final R indexes [all data]	R_1 = 0.1064, wR_2 = 0.2547
Largest diff. peak/hole / e Å^{-3}	0.51/-1.08

Table S2 Fractional Atomic Coordinates (×10^4) and Equivalent Isotropic Displacement Parameters (Å^2×10^3) for 3a. U_{eq} is defined as 1/3 of of the trace of the orthogonalised U_{IJ} tensor.

**Atom**	***x***	***y***	***z***	**U(eq)**
Si2	6530.7(5)	4007.1(4)	7117.8(3)	21.50(14)
Si1	8719.6(5)	2527.8(4)	2672.9(3)	21.71(14)
O1	11029.7(14)	1998.7(13)	1340.2(10)	34.1(4)
O2	7428.1(17)	2711.6(14)	8522.2(11)	40.4(4)
C51	6730.7(17)	5400.9(14)	6699.4(12)	21.8(4)
N1	12675.5(18)	-218.4(16)	3117.5(15)	39.5(5)
C1	9944.8(17)	1755.6(15)	2834.2(13)	23.5(4)
C17	7427.0(17)	1732.0(14)	2994.8(13)	24.0(4)
C24	8381.0(17)	3676.4(14)	3299.1(12)	22.8(4)
C37	7873.9(18)	3284.4(16)	7010.6(14)	26.7(4)
C45	5624.9(17)	3434.2(14)	6569.2(12)	22.3(4)
C56	5809.0(19)	6018.2(16)	6611.0(14)	27.8(4)

C46	5879.7(18)	3579.4(16)	5695.5(13)	27.1(4)
C23	7378.2(18)	4241.0(16)	3353.6(13)	26.9(4)
C6	10878.2(18)	1574.7(16)	2161.6(13)	26.0(4)
C22	6902.8(18)	1463.2(15)	3845.6(14)	27.9(4)
C52	7751.7(19)	5882.2(16)	6542.7(15)	30.2(4)
C55	5885(2)	7082.6(17)	6402.3(15)	31.6(5)
C50	4726.4(18)	2809.5(15)	7008.2(13)	27.1(4)
N2	10325(2)	3775(2)	-1080.2(14)	51.7(6)
C48	4383(2)	2477.3(17)	5718.0(15)	32.1(5)
C29	5880.9(19)	3834.9(16)	8255.9(13)	27.9(4)
C9	9185.4(18)	2892.1(16)	1534.4(13)	27.1(4)
C49	4108(2)	2331.2(16)	6583.2(15)	31.5(5)
C3	10854.9(19)	593.9(16)	3723.1(15)	30.2(4)
C25	9120(2)	4032.6(17)	3690.1(15)	31.3(4)
C2	9982.0(18)	1241.9(16)	3610.8(14)	27.8(4)
C38	8120(2)	2743.3(16)	7705.0(15)	31.1(5)
C10	10245.3(19)	2608.7(16)	1045.0(13)	28.7(4)
C47	5269(2)	3106.1(18)	5272.4(14)	32.2(5)
C28	7124(2)	5137.1(17)	3767.0(15)	32.0(5)
C21	6001(2)	804.1(17)	4110.2(16)	34.5(5)
C4	11787.0(19)	418.3(16)	3026.9(16)	30.9(4)
C18	7002(2)	1325.2(18)	2413.7(16)	32.3(5)
C42	8699.1(19)	3204.9(18)	6232.4(16)	33.3(5)
C53	7839(2)	6950.9(18)	6319.7(17)	37.0(5)
C11	10649(2)	2910.9(19)	191.2(15)	35.3(5)
C30	4828(2)	4270.9(19)	8653.9(15)	36.7(5)
C54	6896(2)	7547.1(17)	6260.7(15)	33.9(5)
C14	8521(2)	3506.8(19)	1098.8(15)	35.2(5)
C12	9959(2)	3509.4(19)	-231.5(15)	38.6(5)
C19	6088(2)	672.5(19)	2673.1(18)	38.4(5)
C34	6393(2)	3204.2(17)	8780.3(14)	32.2(5)
C20	5592(2)	410.8(17)	3521.4(19)	38.5(6)
N4	10900(2)	1522.0(19)	6790(2)	58.8(8)
C5	11782.2(19)	923.9(17)	2245.5(15)	31.4(5)
C26	8875(2)	4925.6(18)	4106.6(16)	36.1(5)
C7	12614(2)	-805.5(19)	3916(2)	42.6(6)
N3	4280(3)	3160(3)	10849.8(15)	70.9(9)
C27	7878(2)	5477.0(17)	4142.7(15)	35.0(5)
C41	9687(2)	2625.9(19)	6146(2)	41.8(6)
C40	9912(2)	2089.6(18)	6857(2)	43.0(6)

C39	9115(2)	2155.8(18)	7637(2)	42.5(6)
C33	5884(3)	3004(2)	9639.2(15)	43.8(6)
C13	8885(2)	3809(2)	249.7(16)	40.6(6)
C31	4297(3)	4075(2)	9502.8(16)	45.4(6)
C8	13515(2)	-523(2)	2379(2)	44.1(6)
C32	4822(3)	3411(2)	10011.2(15)	48.6(7)
C16	9653(3)	4481(2)	-1486.0(18)	56.6(8)
C15	11473(3)	3553(3)	-1553.0(18)	61.2(9)
C44	11596(2)	1277(2)	5974(3)	68.6(12)
C43	11038(3)	853(2)	7510(3)	68.1(11)
C35	3200(4)	3645(3)	11236(2)	74.4(12)
C36	4867(5)	2539(4)	11371(2)	87.2(15)

Table S3 Anisotropic Displacement Parameters (Å^2×10^3) for 3a. The Anisotropic displacement factor exponent takes the form: -2π^2[h^{2}a*^{2}U$_{11}$+2hka*b*U$_{12}$+⋯].

Atom	U_{11}	U_{22}	U_{33}	U_{23}	U_{13}	U_{12}
Si2	22.7(3)	19.3(2)	23.4(3)	0.06(18)	-9.3(2)	1.88(19)
Si1	18.9(3)	19.4(2)	25.5(3)	0.40(19)	-5.4(2)	0.54(19)
O1	27.7(8)	40.6(9)	28.0(8)	0.3(6)	-1.3(6)	6.9(7)
O2	51.2(11)	38.2(9)	37.4(9)	3.1(7)	-25.6(8)	7.7(8)
C51	22.8(9)	20.6(8)	23.3(8)	-2.3(6)	-9.3(7)	3.0(7)
N1	32.3(11)	32.4(10)	54.6(13)	-1.3(9)	-17.0(10)	11.2(8)
C1	21.7(9)	20.2(8)	27.8(9)	-1.4(7)	-6.5(7)	1.1(7)
C17	22.1(9)	16.8(8)	32.1(10)	-0.8(7)	-7.1(8)	1.7(7)
C24	22.0(9)	19.0(8)	25.3(9)	2.3(6)	-4.9(7)	-0.8(7)
C37	23.6(10)	22.7(9)	36.3(10)	-1.2(7)	-13.5(8)	1.1(7)
C45	22.4(9)	18.8(8)	26.3(9)	-1.1(7)	-9.2(7)	2.8(7)
C56	28.5(10)	22.5(9)	35.9(10)	-2.6(7)	-15.6(9)	3.0(8)
C46	26.6(10)	29.1(10)	25.8(9)	-2.2(7)	-7.5(8)	-2.7(8)
C23	24.4(10)	25.9(9)	29.1(9)	-0.2(7)	-7.3(8)	2.0(8)
C6	22.4(10)	25.7(9)	29.4(10)	-1.4(7)	-6.9(8)	0.6(7)
C22	25.9(10)	21.3(9)	33.8(10)	2.3(7)	-6.0(8)	0.2(7)
C52	27.5(11)	23.9(9)	39.6(11)	0.6(8)	-11.8(9)	0.2(8)
C55	38.3(12)	25.0(9)	37.6(11)	-3.3(8)	-21.6(10)	7.4(8)
C50	30.1(11)	21.9(9)	28.7(9)	1.3(7)	-9.0(8)	-0.7(8)
N2	70.3(17)	53.9(14)	27.3(10)	8.4(9)	-10.2(11)	-14.6(13)
C48	31.8(11)	28.2(10)	39.9(12)	-12.4(9)	-12.9(9)	0.4(8)
C29	32.8(11)	25.4(9)	26.9(9)	-0.4(7)	-11.4(8)	-1.5(8)
C9	27.6(10)	24.9(9)	27.4(9)	0.4(7)	-6.8(8)	-0.7(8)
C49	31.5(11)	21.5(9)	41.7(12)	-1.9(8)	-10.5(9)	-3.7(8)

C3	27.7(11)	24.9(9)	37.9(11)	2.6(8)	-11.8(9)	1.3(8)
C25	29.3(11)	27.1(10)	40.5(12)	-2.2(8)	-15.2(9)	-0.7(8)
C2	25.5(10)	25.1(9)	31.2(10)	1.7(7)	-7.2(8)	0.6(8)
C38	34.5(12)	23.3(9)	41.6(12)	-2.3(8)	-21.2(10)	1.5(8)
C10	30.8(11)	25.7(9)	27.8(10)	-0.5(7)	-6.1(8)	-1.2(8)
C47	34.1(12)	34.8(11)	29.7(10)	-9.3(8)	-9.8(9)	-1.1(9)
C28	32.2(11)	25.6(9)	36.5(11)	-2.2(8)	-8.8(9)	6.3(8)
C21	27.4(11)	24.1(10)	45.4(13)	6.7(9)	-3.9(9)	-1.2(8)
C4	27.6(11)	20.9(9)	45.7(12)	-2.9(8)	-13.9(9)	3.6(8)
C18	29.6(11)	29.7(10)	39.4(11)	-6.5(9)	-11.2(9)	-1.5(8)
C42	24.0(10)	28.0(10)	45.9(13)	-3.7(9)	-7.5(9)	2.6(8)
C53	34.4(12)	25.5(10)	51.8(14)	2.9(9)	-15.0(11)	-5.5(9)
C11	37.2(13)	37.5(12)	27.5(10)	-1.1(9)	-3.5(9)	-4.1(10)
C30	40.9(13)	36.8(12)	30.3(11)	-4.6(9)	-7.0(10)	2.1(10)
C54	45.4(14)	20.7(9)	40.0(12)	0.9(8)	-20.4(10)	-1.1(9)
C14	37.9(13)	33.4(11)	33.3(11)	4.0(9)	-12.1(10)	2.3(9)
C12	53.6(16)	34.8(11)	26.7(10)	3.4(8)	-11.1(10)	-10.0(11)
C19	31.9(12)	32.4(11)	55.8(15)	-11.8(10)	-17.3(11)	-1.6(9)
C34	46.2(13)	27.2(10)	27.4(10)	-1.3(8)	-17.5(9)	-2.5(9)
C20	26.8(11)	18.8(9)	67.6(17)	-0.3(10)	-10.7(11)	-2.6(8)
N4	39.6(13)	32.6(11)	111(2)	-10.8(13)	-33.2(15)	13.4(10)
C5	24.7(10)	29.4(10)	38.0(11)	-4.8(8)	-6.4(9)	5.5(8)
C26	40.3(13)	28.8(10)	43.7(13)	-5.5(9)	-17.9(11)	-3.6(9)
C7	38.2(13)	24.4(10)	69.7(18)	5.2(11)	-26.3(13)	1.5(9)
N3	105(3)	72.3(19)	23.2(11)	-3.5(11)	1.4(13)	-12.0(18)
C27	44.6(14)	22.3(9)	37.9(11)	-5.0(8)	-11.2(10)	1.2(9)
C41	27.8(12)	29.0(11)	66.3(17)	-11.4(11)	-8.8(11)	5.5(9)
C40	30.8(12)	24.0(10)	80(2)	-10.0(11)	-24.5(13)	5.4(9)
C39	48.0(15)	23.6(10)	69.1(18)	-3.1(10)	-39.2(14)	5.4(10)
C33	70.0(19)	38.6(12)	25.9(11)	1.7(9)	-19.3(12)	-7.2(12)
C13	51.2(15)	37.4(12)	34.6(12)	8.2(9)	-18.4(11)	-3.7(11)
C31	49.9(16)	48.7(15)	31.7(12)	-9.1(10)	0.1(11)	-5.5(12)
C8	30.5(12)	32.3(12)	69.0(18)	-7.4(11)	-14.9(12)	10.9(10)
C32	77(2)	43.8(14)	24.0(11)	-4.2(9)	-8.9(12)	-18.1(14)
C16	95(3)	42.9(14)	37.4(13)	11.1(11)	-29.3(15)	-22.8(16)
C15	80(2)	65(2)	28.2(12)	4.1(12)	1.0(14)	-18.9(18)
C44	27.4(14)	34.5(14)	139(4)	-18.5(18)	-15.0(18)	9.1(11)
C43	53.2(19)	32.8(13)	134(4)	-3.2(17)	-57(2)	13.6(13)
C35	99(3)	81(2)	33.2(14)	-19.0(15)	9.5(16)	-41(2)
C36	146(4)	87(3)	25.1(14)	7.3(15)	-22(2)	-16(3)

Table S4 Bond Lengths for 3a.

Atom	Atom	Length/Å	Atom	Atom	Length/Å
Si2	C51	1.873(2)	N2	C16	1.452(4)
Si2	C37	1.843(2)	N2	C15	1.450(5)
Si2	C45	1.877(2)	C48	C49	1.381(3)
Si2	C29	1.834(2)	C48	C47	1.392(3)
Si1	C1	1.847(2)	C29	C30	1.405(3)
Si1	C17	1.878(2)	C29	C34	1.398(3)
Si1	C24	1.875(2)	C9	C10	1.390(3)
Si1	C9	1.839(2)	C9	C14	1.414(3)
O1	C6	1.387(3)	C3	C2	1.380(3)
O1	C10	1.390(3)	C3	C4	1.414(3)
O2	C38	1.386(3)	C25	C26	1.392(3)
O2	C34	1.379(3)	C38	C39	1.401(3)
C51	C56	1.396(3)	C10	C11	1.390(3)
C51	C52	1.394(3)	C28	C27	1.388(3)
N1	C4	1.377(3)	C21	C20	1.388(4)
N1	C7	1.448(4)	C4	C5	1.399(3)
N1	C8	1.444(4)	C18	C19	1.397(3)
C1	C6	1.393(3)	C42	C41	1.384(3)
C1	C2	1.406(3)	C53	C54	1.391(3)
C17	C22	1.398(3)	C11	C12	1.414(4)
C17	C18	1.396(3)	C30	C31	1.383(3)
C24	C23	1.396(3)	C14	C13	1.379(3)
C24	C25	1.399(3)	C12	C13	1.405(4)
C37	C38	1.393(3)	C19	C20	1.386(4)
C37	C42	1.415(3)	C34	C33	1.394(3)
C45	C46	1.397(3)	N4	C40	1.380(3)
C45	C50	1.397(3)	N4	C44	1.447(5)
C56	C55	1.391(3)	N4	C43	1.452(5)
C46	C47	1.387(3)	C26	C27	1.387(4)
C23	C28	1.393(3)	N3	C32	1.381(3)
C6	C5	1.400(3)	N3	C35	1.453(6)
C22	C21	1.391(3)	N3	C36	1.447(6)
C52	C53	1.401(3)	C41	C40	1.403(4)
C55	C54	1.376(3)	C40	C39	1.399(4)
C50	C49	1.399(3)	C33	C32	1.389(5)
N2	C12	1.370(3)	C31	C32	1.412(4)

Table S5 Bond Angles for 3a.

Atom	Atom	Atom	Angle/°	Atom	Atom	Atom	Angle/°
C51	Si2	C45	107.90(8)	C10	C9	C14	115.2(2)
C37	Si2	C51	112.34(9)	C14	C9	Si1	123.13(17)
C37	Si2	C45	112.36(9)	C48	C49	C50	119.9(2)
C29	Si2	C51	112.83(9)	C2	C3	C4	119.6(2)
C29	Si2	C37	101.74(10)	C26	C25	C24	121.3(2)
C29	Si2	C45	109.65(10)	C3	C2	C1	124.2(2)
C1	Si1	C17	111.15(9)	O2	C38	C37	125.0(2)
C1	Si1	C24	112.77(9)	O2	C38	C39	112.8(2)
C24	Si1	C17	106.55(9)	C37	C38	C39	122.2(2)
C9	Si1	C1	101.39(10)	O1	C10	C9	124.88(19)
C9	Si1	C17	112.21(10)	C11	C10	O1	111.7(2)
C9	Si1	C24	112.88(9)	C11	C10	C9	123.4(2)
C6	O1	C10	125.60(17)	C46	C47	C48	120.1(2)
C34	O2	C38	125.89(17)	C27	C28	C23	119.5(2)
C56	C51	Si2	119.61(15)	C20	C21	C22	119.8(2)
C52	C51	Si2	122.45(15)	N1	C4	C3	121.1(2)
C52	C51	C56	117.66(18)	N1	C4	C5	121.2(2)
C4	N1	C7	120.0(2)	C5	C4	C3	117.7(2)
C4	N1	C8	119.4(2)	C17	C18	C19	121.2(2)
C8	N1	C7	118.4(2)	C41	C42	C37	123.4(2)
C6	C1	Si1	121.07(15)	C54	C53	C52	119.9(2)
C6	C1	C2	114.90(18)	C10	C11	C12	120.2(2)
C2	C1	Si1	123.84(16)	C31	C30	C29	123.3(2)
C22	C17	Si1	119.78(16)	C55	C54	C53	120.0(2)
C18	C17	Si1	122.53(17)	C13	C14	C9	123.2(2)
C18	C17	C22	117.52(19)	N2	C12	C11	121.0(3)
C23	C24	Si1	119.73(15)	N2	C12	C13	121.5(3)
C23	C24	C25	117.51(19)	C13	C12	C11	117.5(2)
C25	C24	Si1	122.69(16)	C20	C19	C18	120.0(2)
C38	C37	Si2	121.02(18)	O2	C34	C29	125.1(2)
C38	C37	C42	115.6(2)	O2	C34	C33	112.6(2)
C42	C37	Si2	123.38(16)	C33	C34	C29	122.3(2)
C46	C45	Si2	120.02(15)	C19	C20	C21	119.8(2)
C46	C45	C50	118.19(18)	C40	N4	C44	119.8(3)
C50	C45	Si2	121.70(15)	C40	N4	C43	119.5(3)
C55	C56	C51	121.8(2)	C44	N4	C43	116.7(3)
C47	C46	C45	121.0(2)	C4	C5	C6	120.7(2)

C28	C23	C24	121.7(2)	C27	C26	C25	119.9(2)
O1	C6	C1	125.17(18)	C32	N3	C35	120.2(3)
O1	C6	C5	111.95(18)	C32	N3	C36	119.5(4)
C1	C6	C5	122.9(2)	C36	N3	C35	119.6(3)
C21	C22	C17	121.7(2)	C26	C27	C28	120.1(2)
C51	C52	C53	120.9(2)	C42	C41	C40	119.8(3)
C54	C55	C56	119.8(2)	N4	C40	C41	120.9(3)
C45	C50	C49	120.9(2)	N4	C40	C39	120.8(3)
C12	N2	C16	120.0(3)	C39	C40	C41	118.2(2)
C12	N2	C15	120.8(3)	C40	C39	C38	120.8(2)
C15	N2	C16	117.9(2)	C32	C33	C34	120.9(3)
C49	C48	C47	119.9(2)	C14	C13	C12	120.5(2)
C30	C29	Si2	123.26(17)	C30	C31	C32	119.7(3)
C34	C29	Si2	121.04(18)	N3	C32	C33	121.3(3)
C34	C29	C30	115.6(2)	N3	C32	C31	120.6(3)
C10	C9	Si1	121.66(16)	C33	C32	C31	118.1(2)

Table S6 Hydrogen Atom Coordinates (Å×10^4) and Isotropic Displacement Parameters (Å^2×10^3) for 3a.

**Atom**	***x***	***y***	***z***	**U(eq)**
H56	5111.06	5702.83	6695.78	33
H46	6481.14	4009.13	5386.1	33
H23	6855.91	4007.99	3102.21	32
H22	7169.12	1737.76	4253.66	34
H52	8396.72	5480.11	6587.81	36
H55	5241.54	7487.2	6357.63	38
H50	4532.5	2708.08	7603.33	32
H48	3968.16	2149.05	5427.64	38
H49	3500.5	1906.54	6889.17	38
H3	10829.66	267.98	4264.69	36
H25	9803.18	3657.42	3670.91	38
H2	9366.56	1347.69	4088.93	33
H47	5454.63	3211.3	4677.67	39
H28	6439.22	5512.44	3791.52	38
H21	5667.07	623.64	4692.2	41
H18	7341.16	1495.39	1830.93	39
H42	8568.18	3568.06	5743.57	40
H53	8541.3	7268.41	6208.83	44
H11	11390.45	2714.77	-109.38	42
H30	4461.87	4722.62	8323.54	44

H54	6950.32	8274.42	6122.72	41
H14	7789.94	3721.77	1405.64	42
H19	5806.87	408.09	2268.32	46
H20	4973.14	-36.37	3699.07	46
H5	12399.08	824.55	1766.83	38
H26	9389.66	5156.76	4365.72	43
H7A	12589.19	-329.06	4336.91	64
H7B	13276.3	-1268.53	3857.21	64
H7C	11935.83	-1215.98	4094.56	64
H27	7710.88	6088.08	4424.85	42
H41	10210.12	2591.64	5608.26	50
H39	9251.17	1797.93	8125.99	51
H33	6268.14	2583.29	9974.83	53
H13	8404.9	4223.61	-11.95	49
H31	3583.19	4386.27	9742.44	54
H8A	13149.16	-820.2	2015.16	66
H8B	14033.34	-1040	2545.97	66
H8C	13932	84.72	2073.84	66
H16A	8915.97	4187.44	-1396.59	85
H16B	10031.32	4585.94	-2089.16	85
H16C	9557.23	5147.06	-1246.03	85
H15A	11985.19	3916.98	-1341.13	92
H15B	11579.92	3785.05	-2147.45	92
H15C	11631.46	2805.45	-1489.86	92
H44A	11158.44	899.79	5705.89	103
H44B	12243.24	847.17	6038.64	103
H44C	11857.21	1919.68	5623.54	103
H43A	10931.82	1262.46	7986.55	102
H43B	11793.65	535	7370.48	102
H43C	10481.31	309.86	7657.83	102
H35A	3288.76	4389.5	11231.68	112
H35B	2909.11	3338.95	11816.63	112
H35C	2673.2	3531.92	10920.76	112
H36A	5080.35	1864.32	11153.01	131
H36B	4375.32	2440.29	11947.44	131
H36C	5542.07	2893.25	11366.13	131

5. NMR spectra

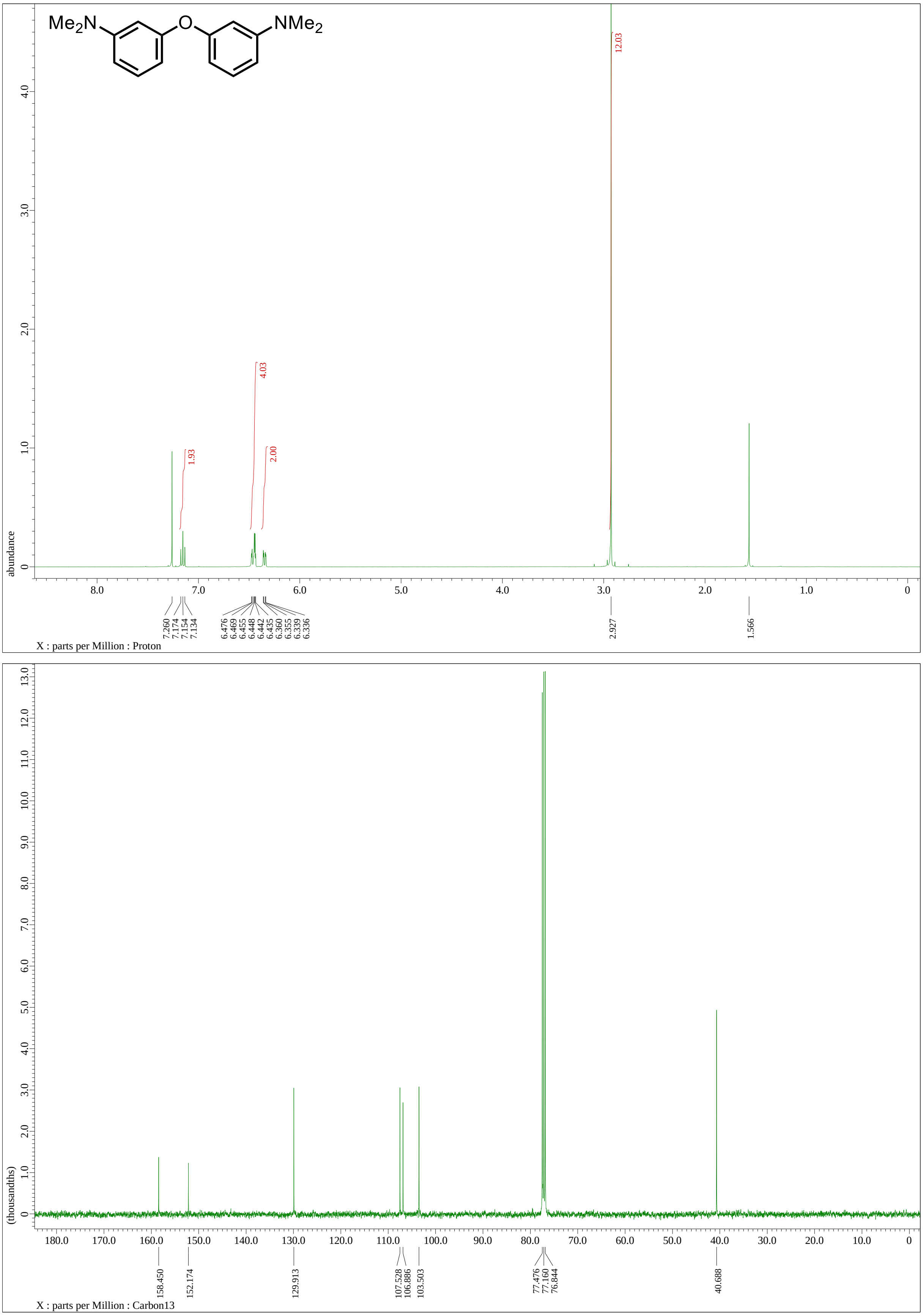

Figure S1: ^{1}H NMR (top) and ^{13}C NMR (bottom) of **1a**.

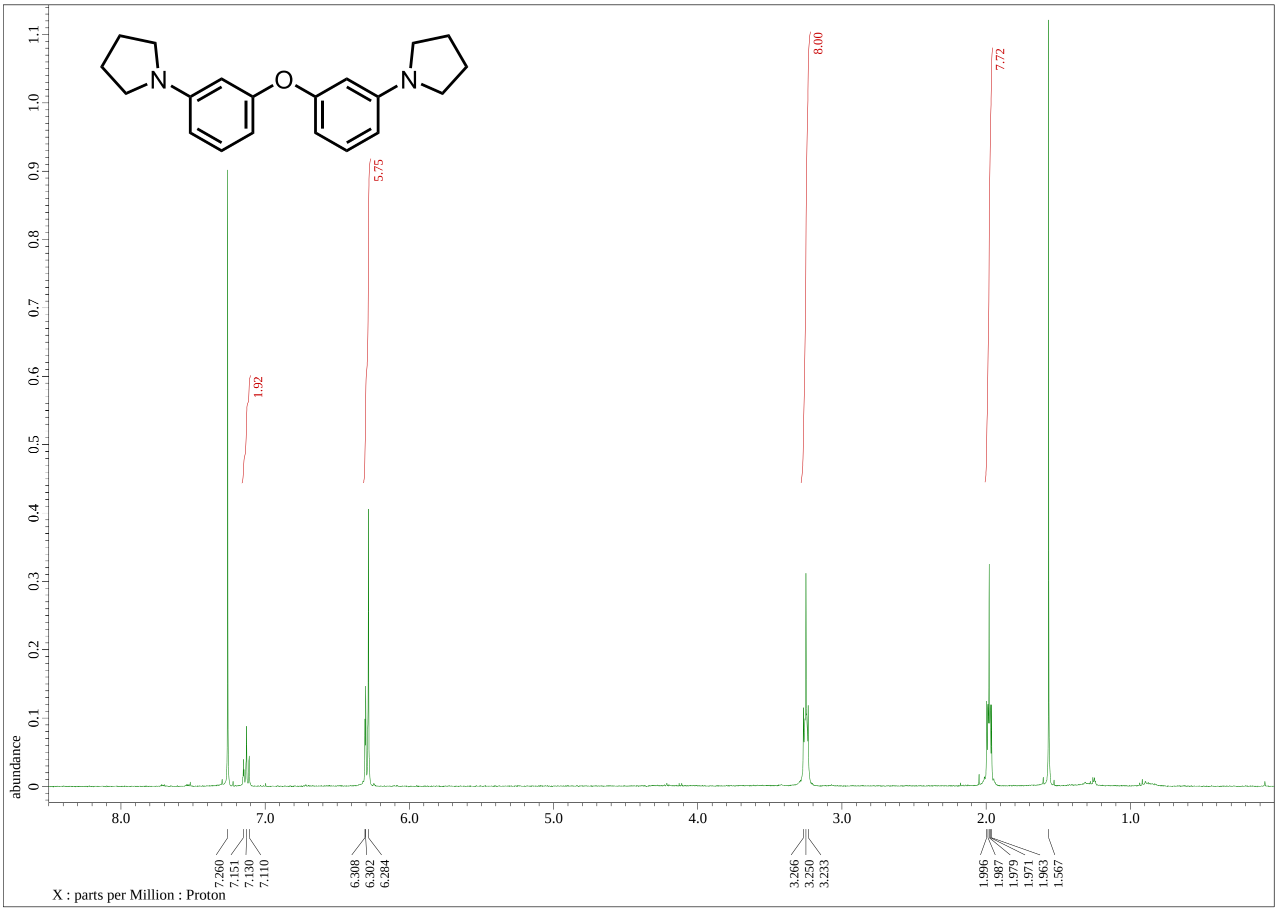

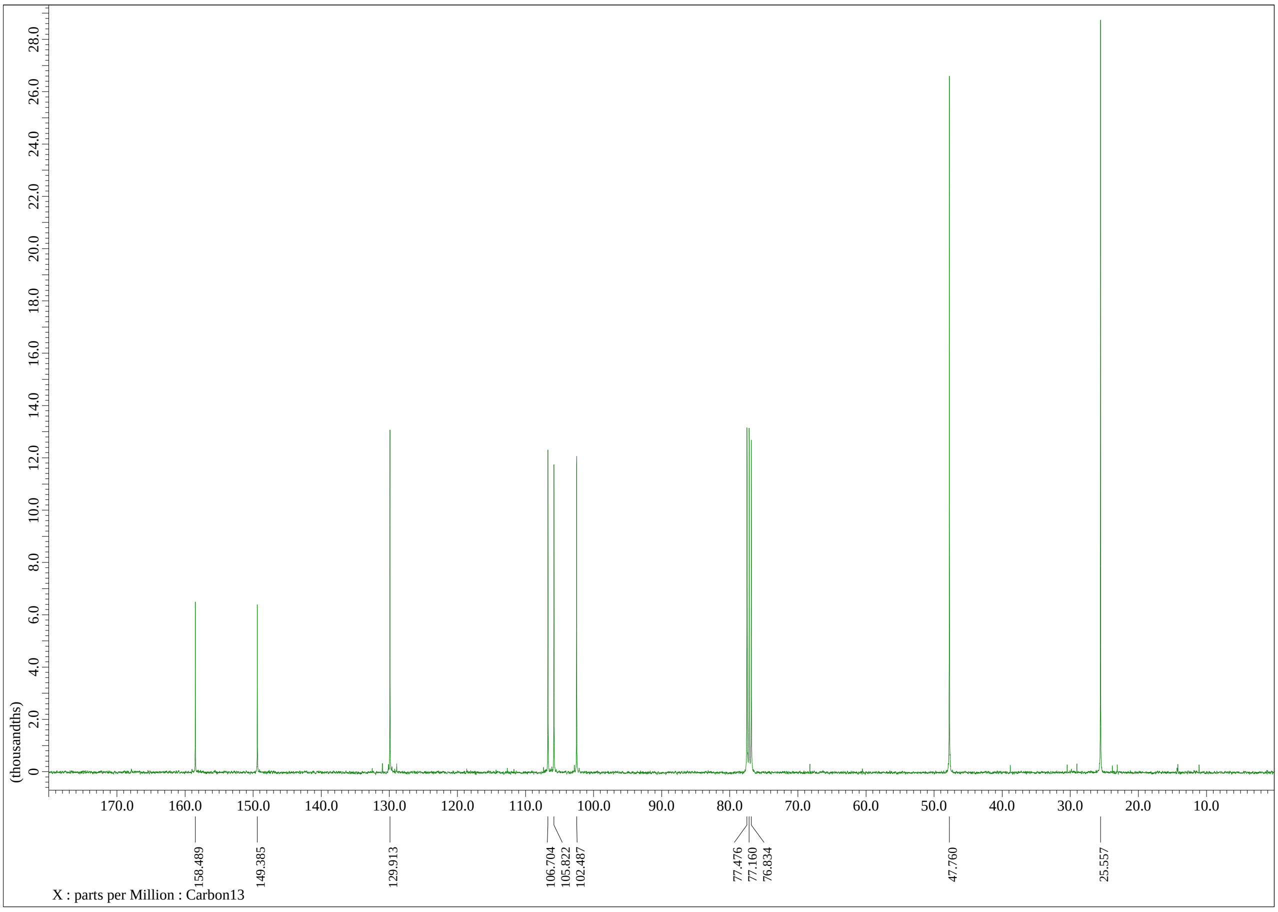

Figure S2: ^{1}H NMR (top) and ^{13}C NMR (bottom) of **1b**.

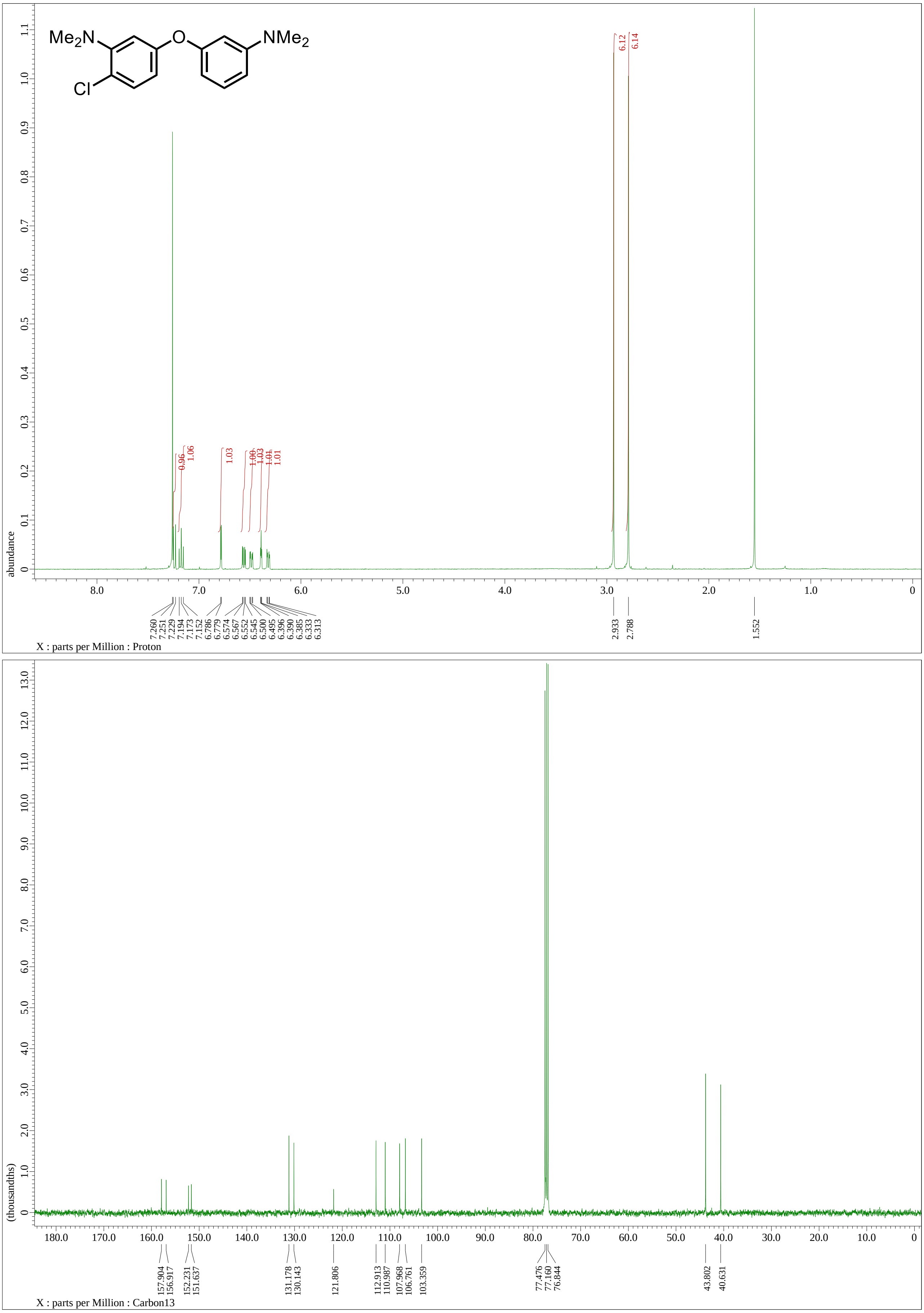

Figure S3: ^{1}H NMR (top) and ^{13}C NMR (bottom) of **1c**.

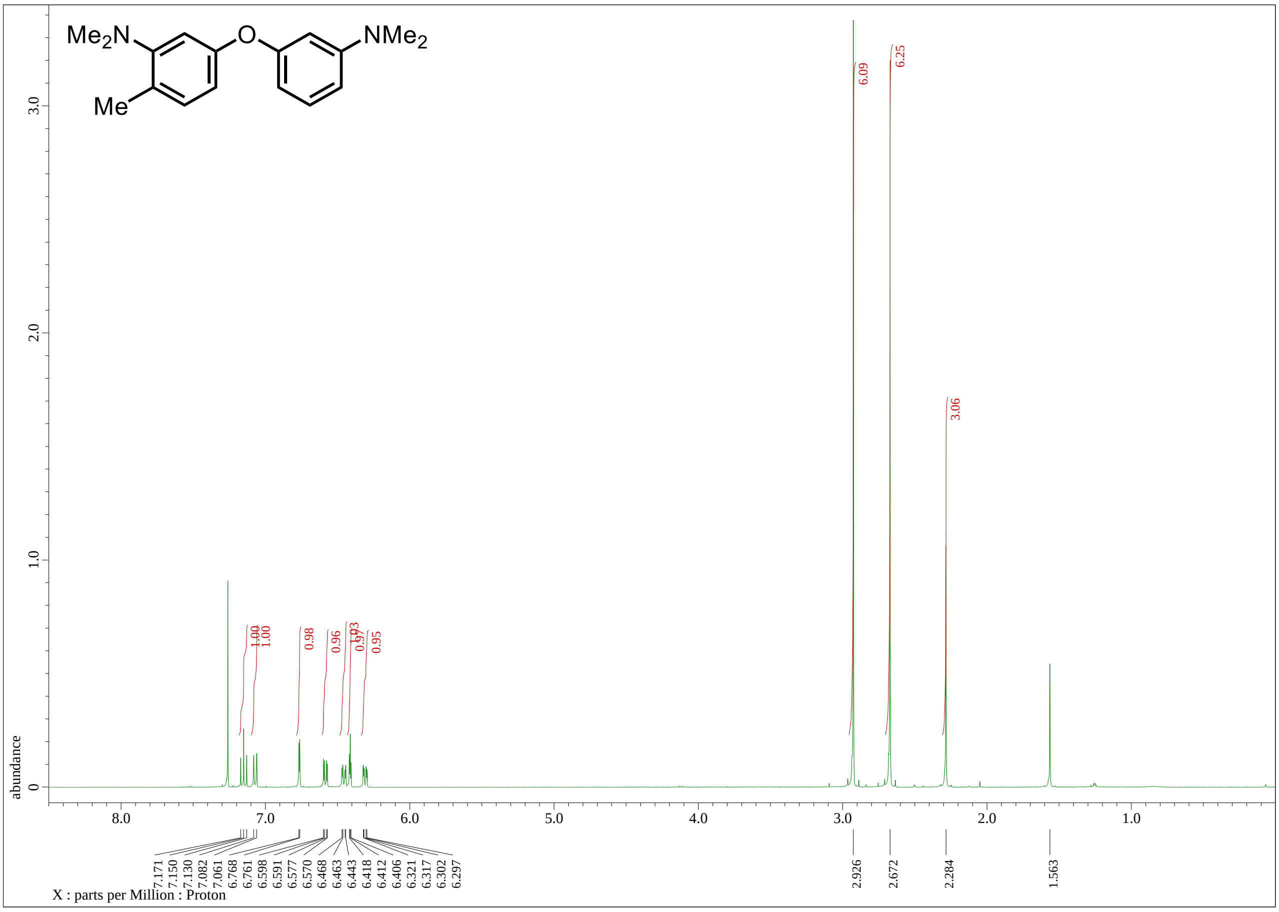

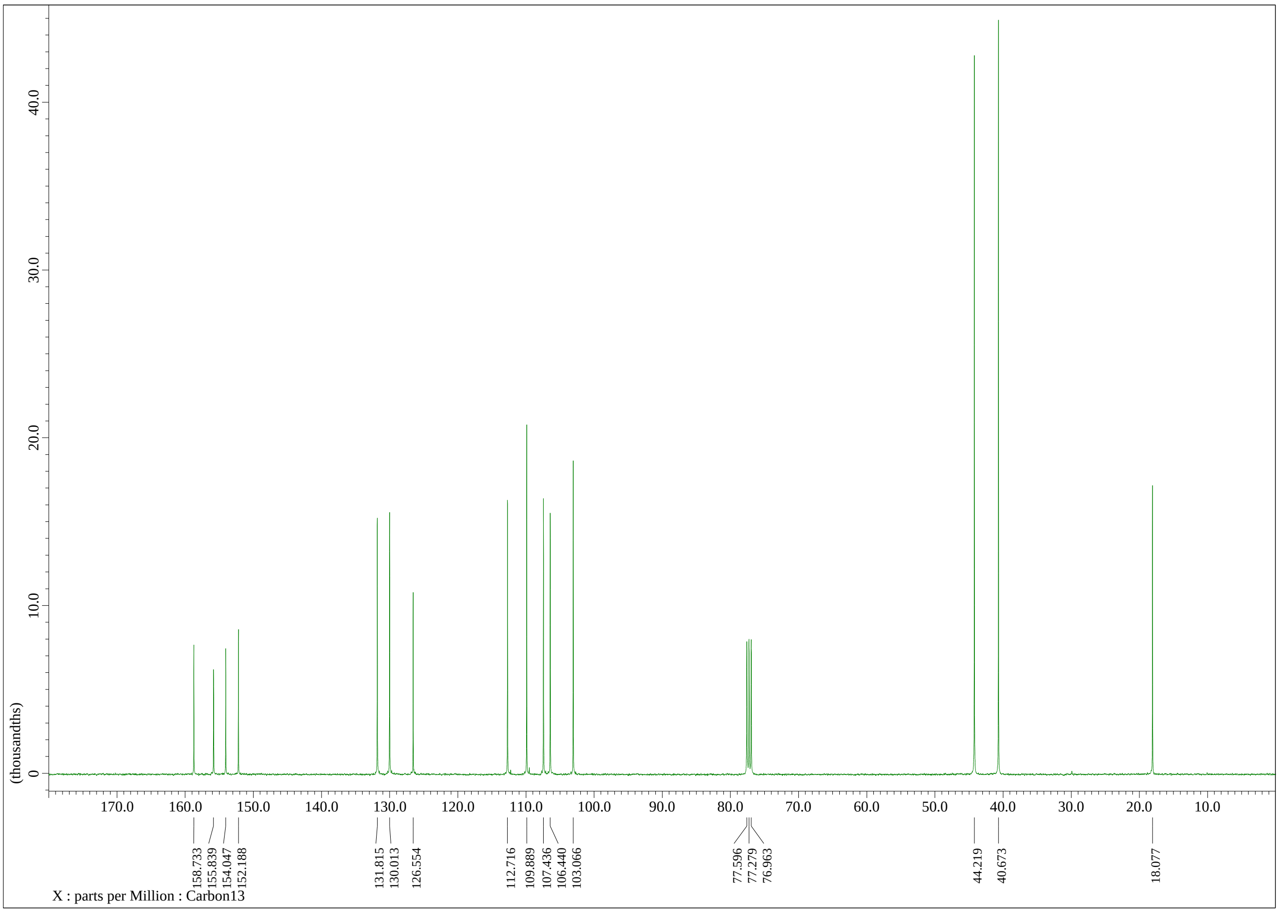

Figure S4: ^{1}H NMR (top) and ^{13}C NMR (bottom) of **1d**.

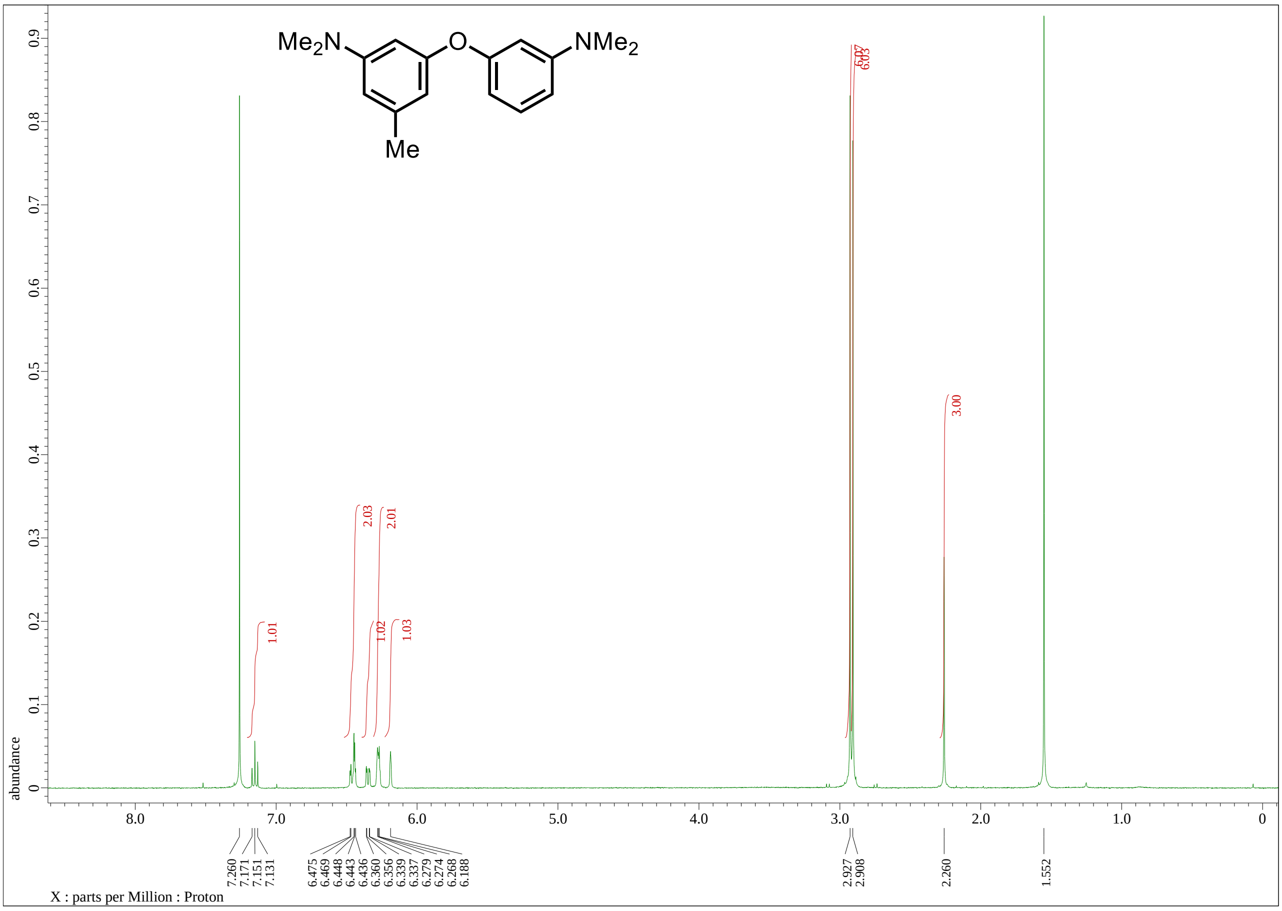

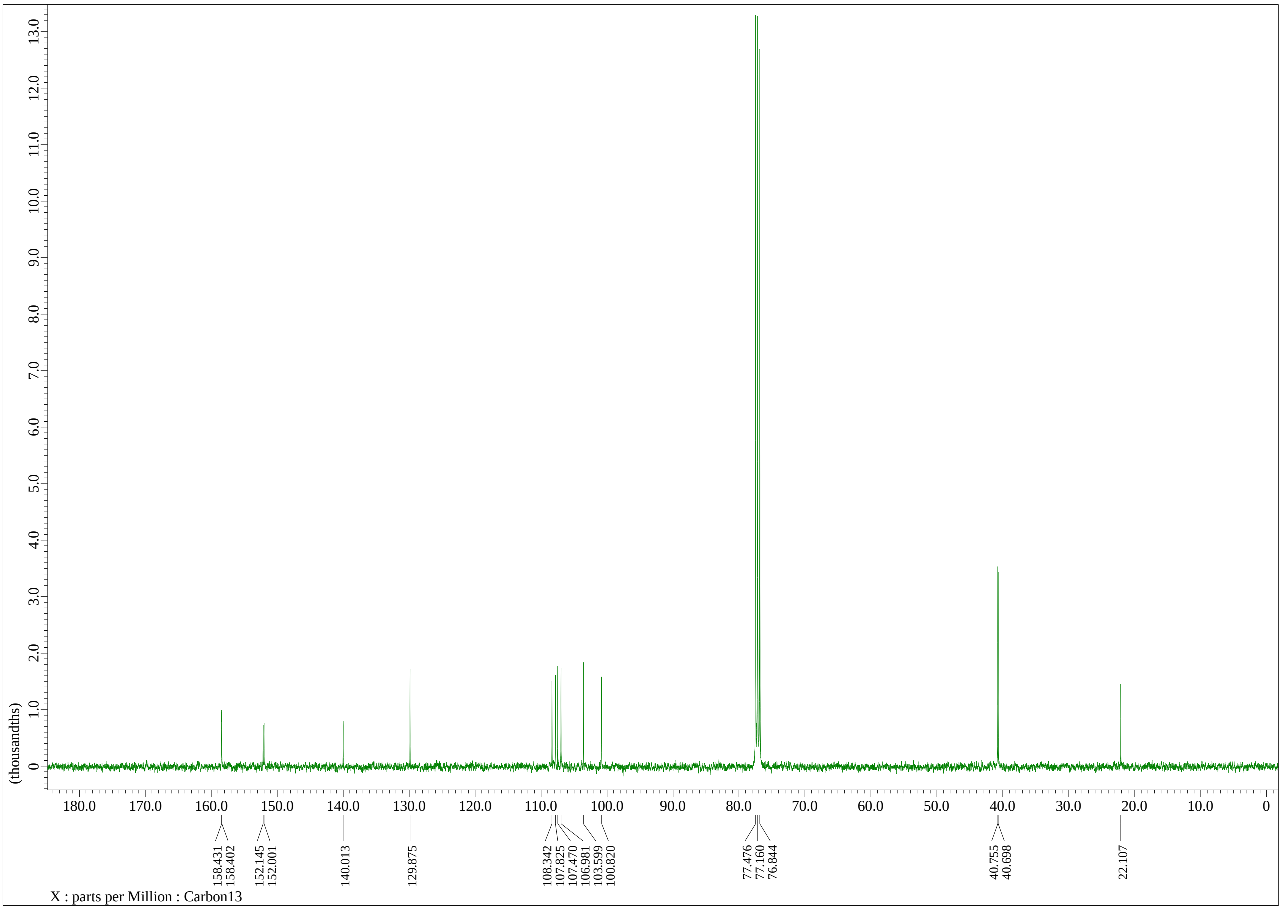

Figure S5: ^{1}H NMR (top) and ^{13}C NMR (bottom) of **1e**.

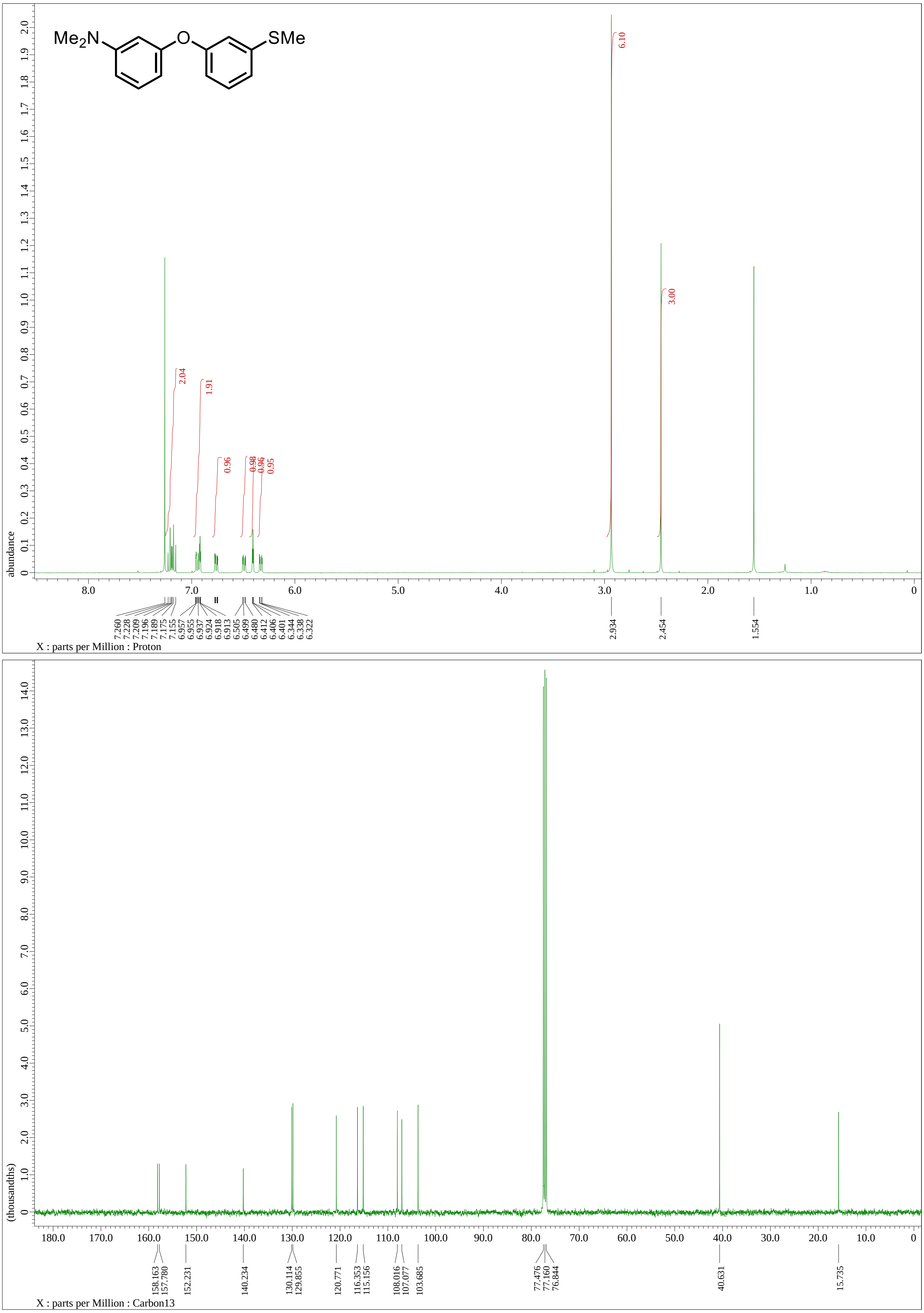

Figure S6: ^{1}H NMR (top) and ^{13}C NMR (bottom) of **1f**.

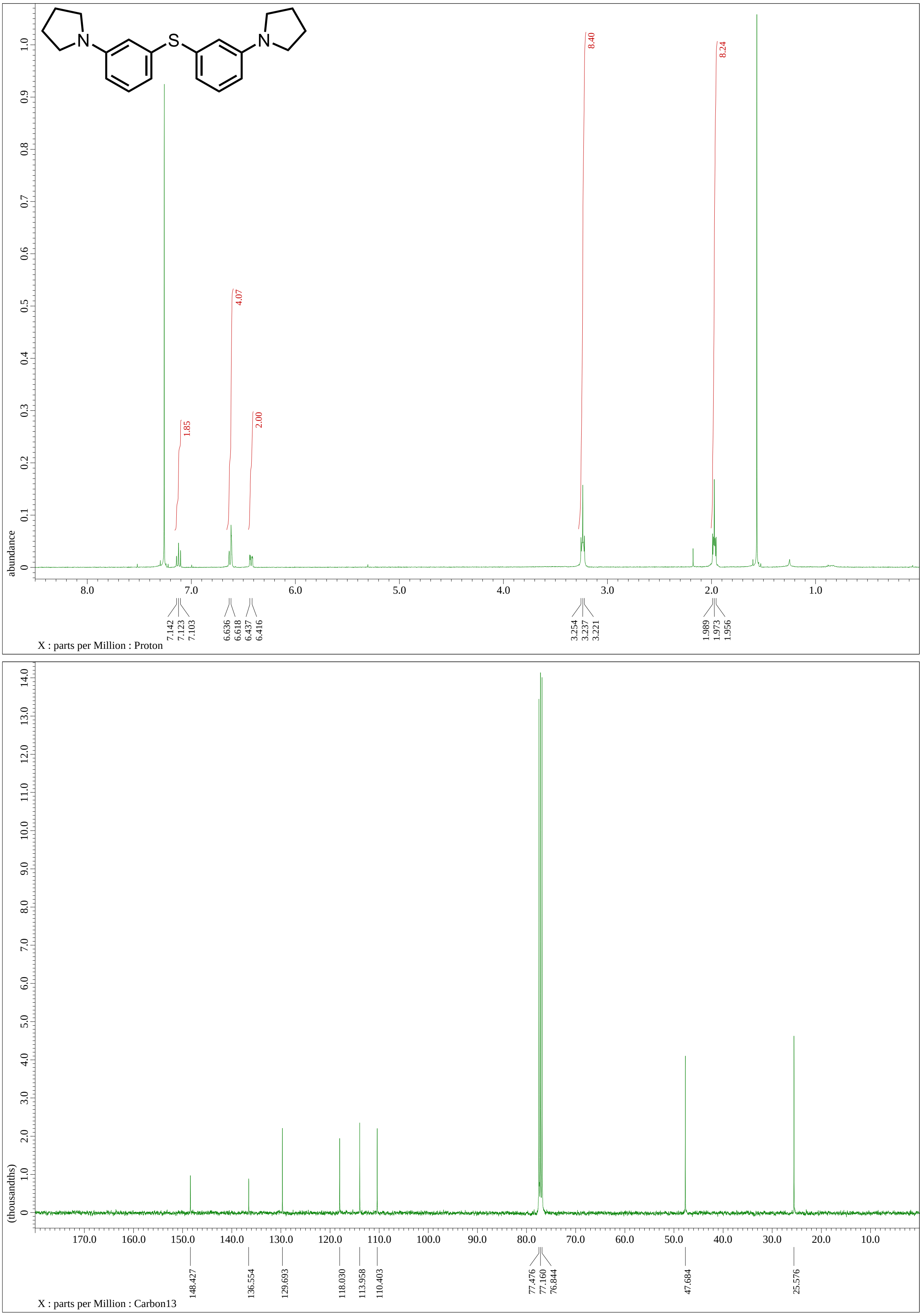

Figure S7: ^{1}H NMR (top) and ^{13}C NMR (bottom) of **1g**

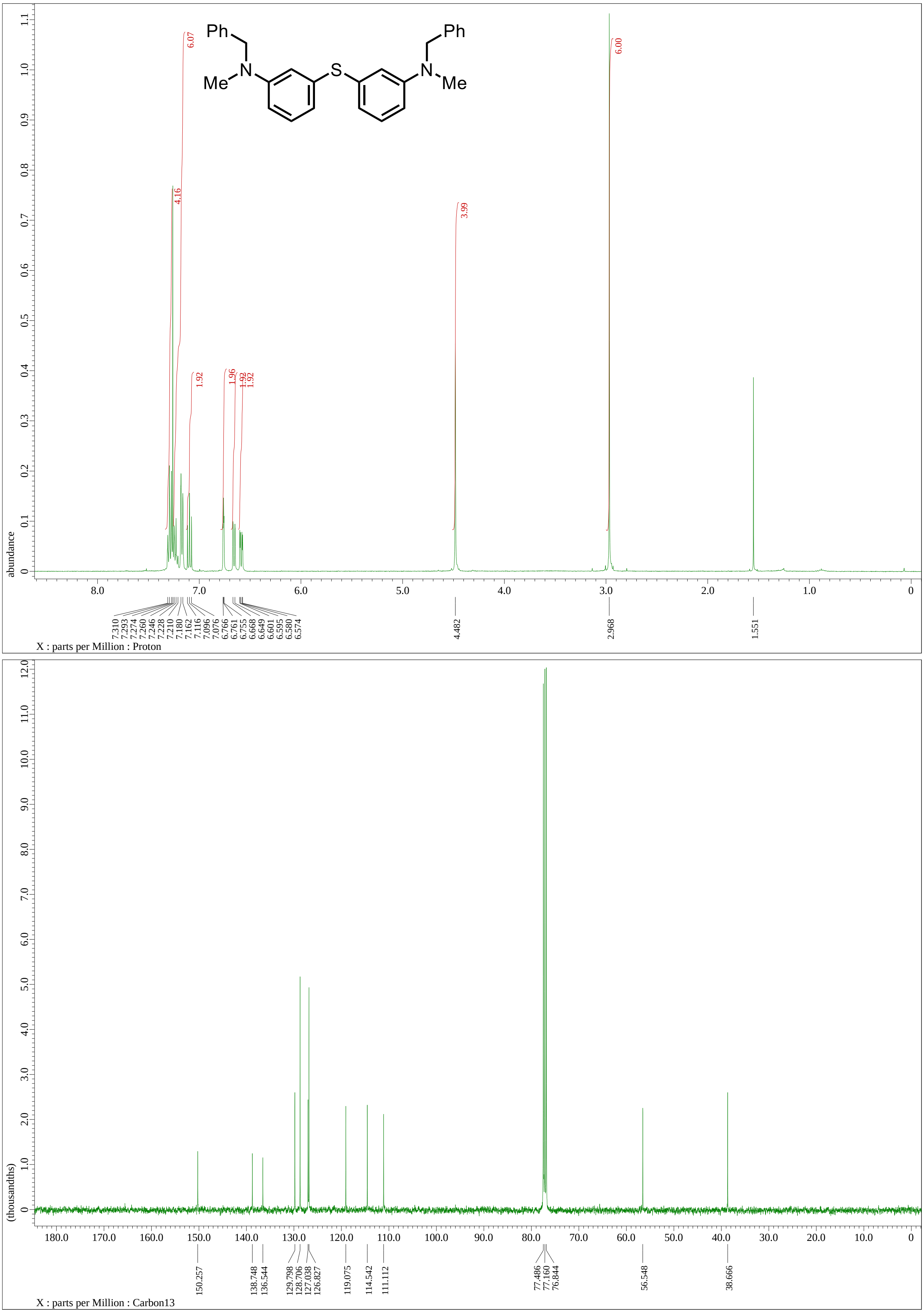

Figure S8: ^{1}H NMR (top) and ^{13}C NMR (bottom) of **1h**.

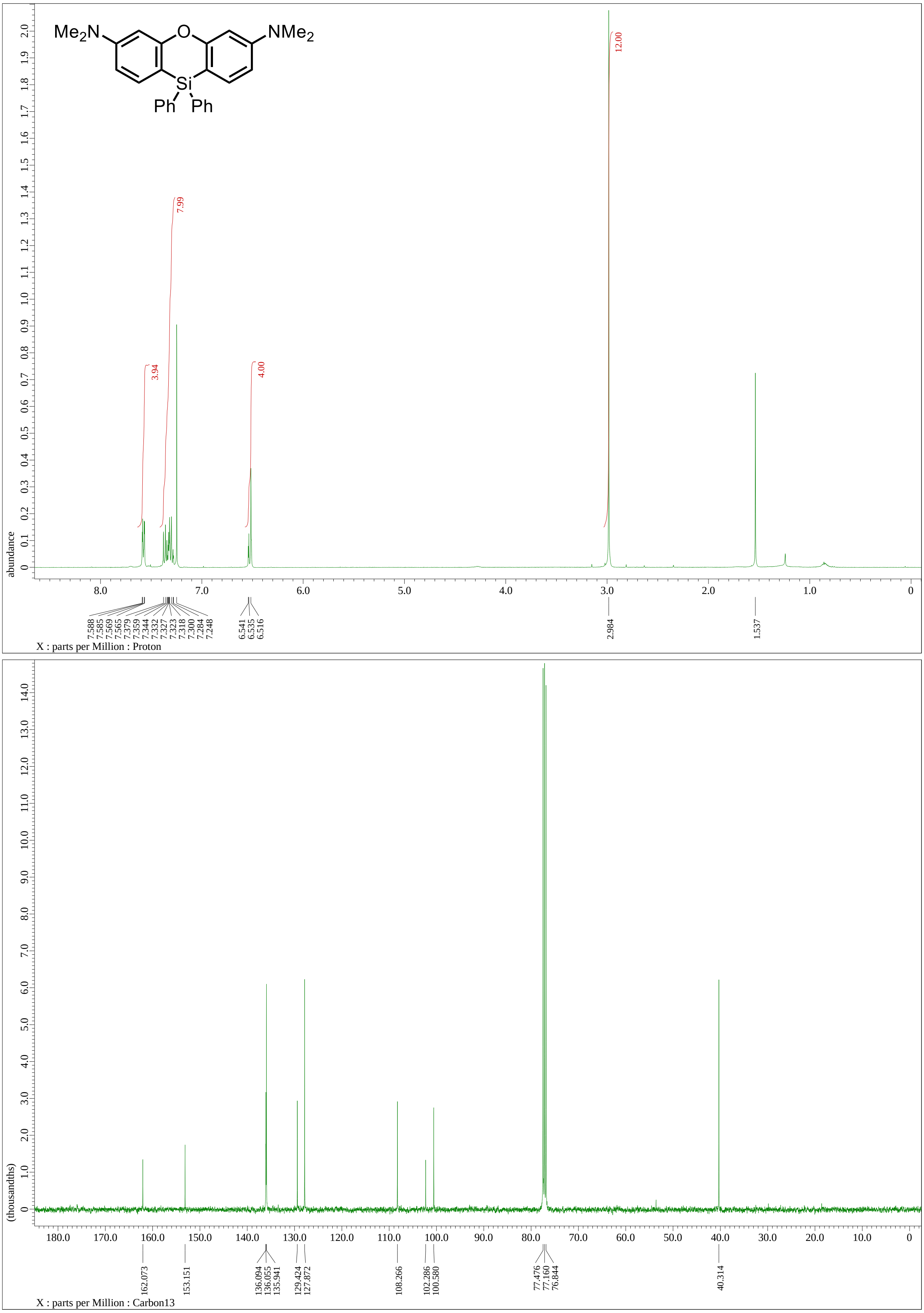

Figure S9: ^{1}H NMR (top) and ^{13}C NMR (bottom) of **3a**.

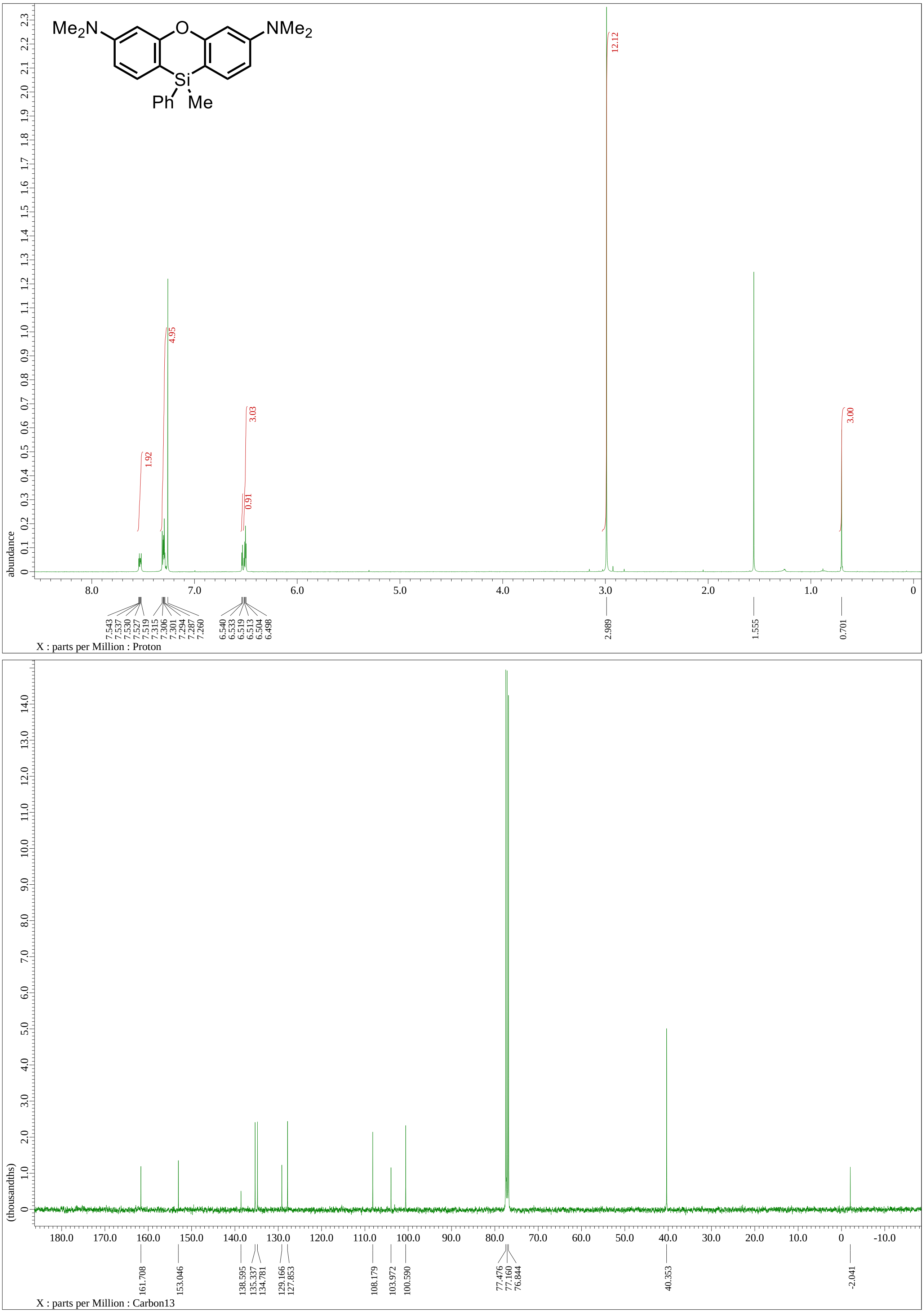

Figure S10: ^{1}H NMR (top) and ^{13}C NMR (bottom) of **3b**.

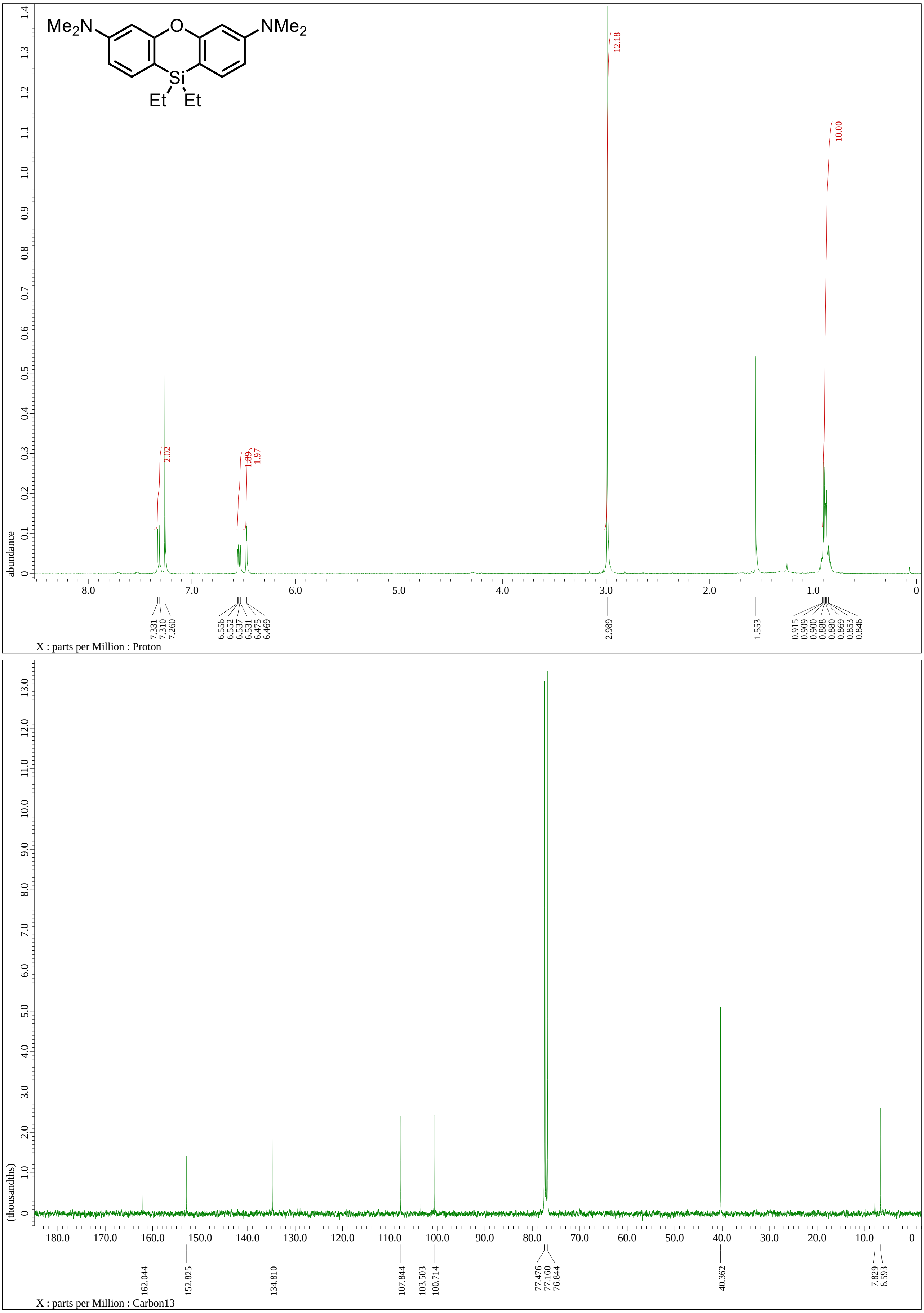

Figure S11: ^{1}H NMR (top) and ^{13}C NMR (bottom) of **3c**.

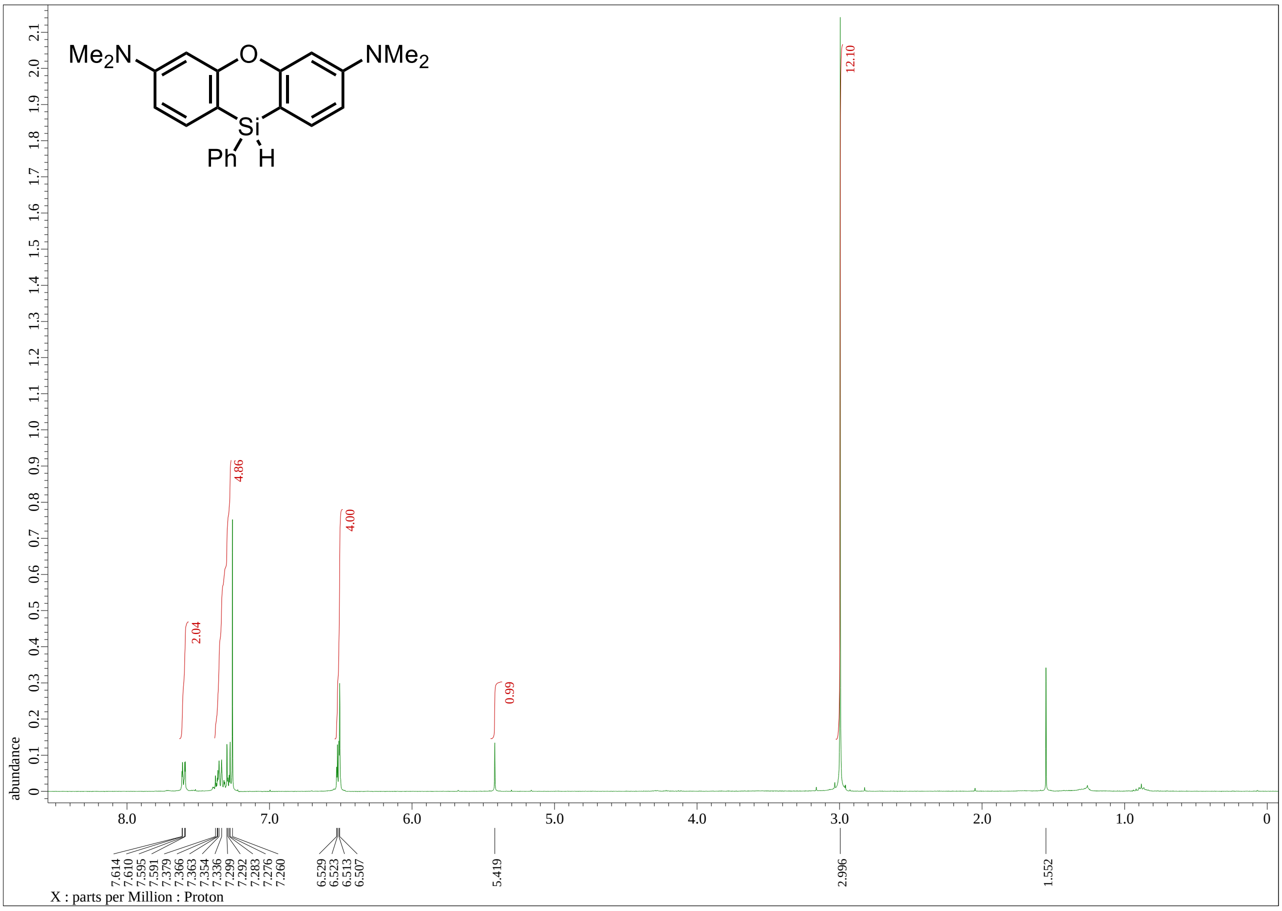

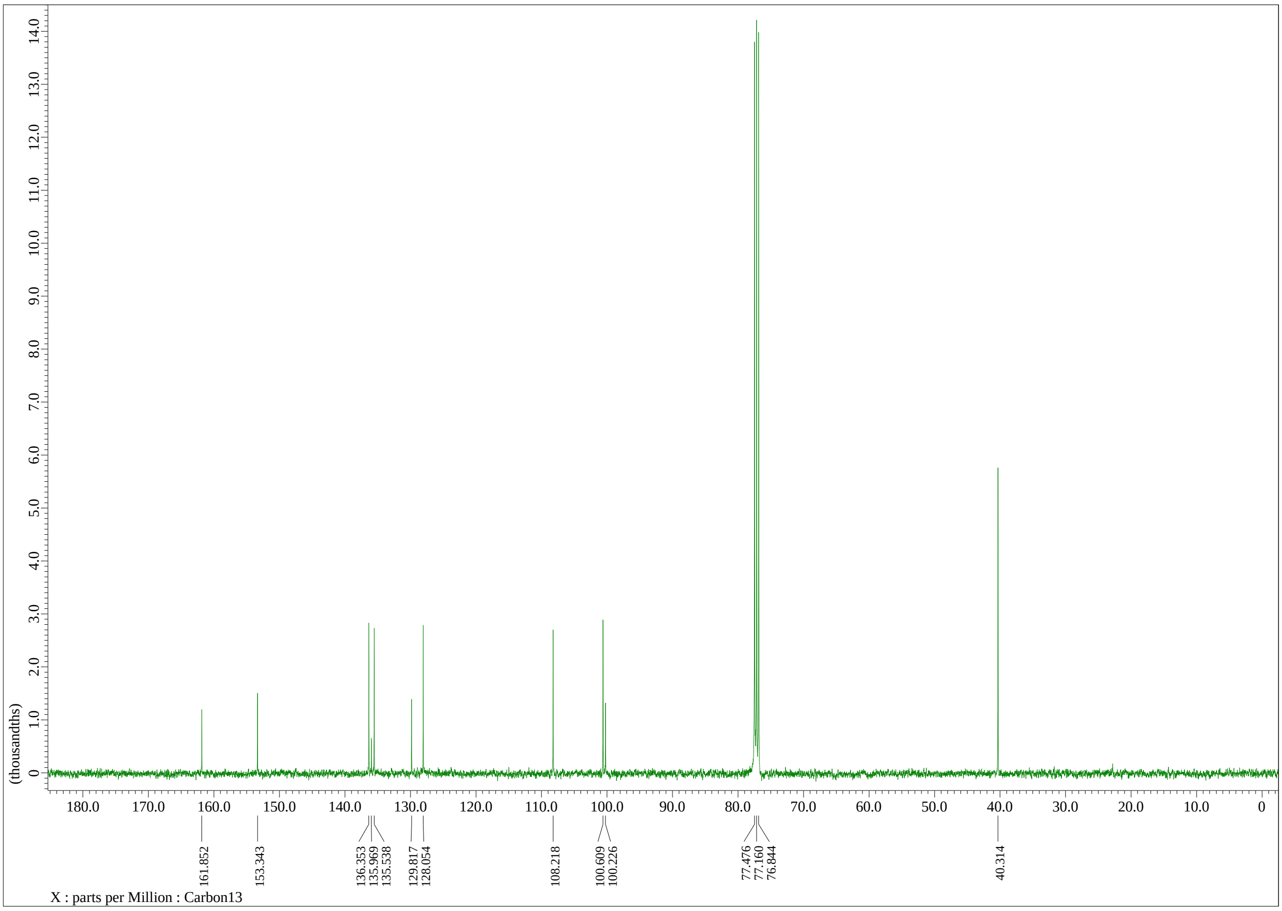

Figure S12: ^{1}H NMR (top) and ^{13}C NMR (bottom) of **3d**.

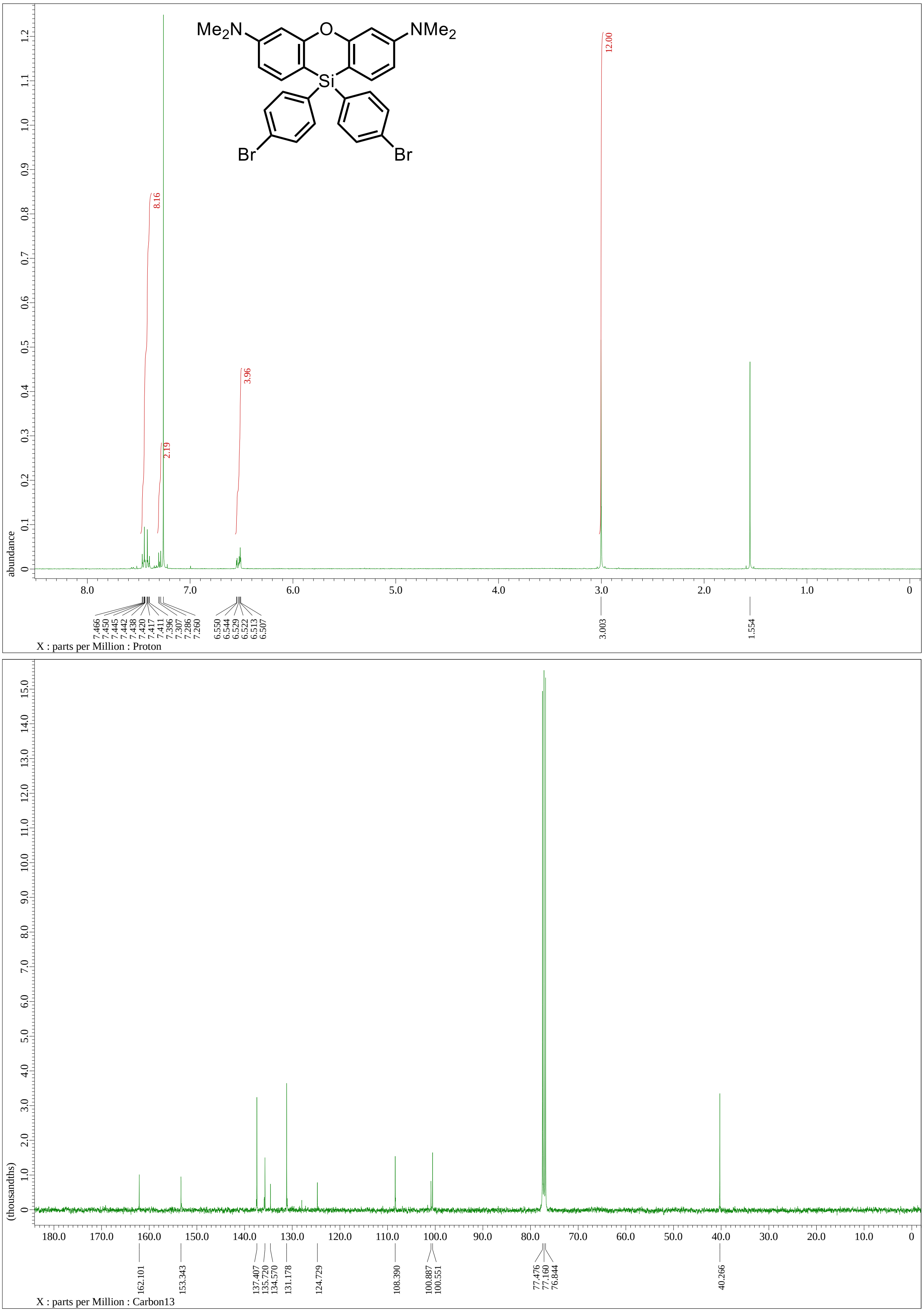

Figure S13: ^{1}H NMR (top) and ^{13}C NMR (bottom) of **3e**.

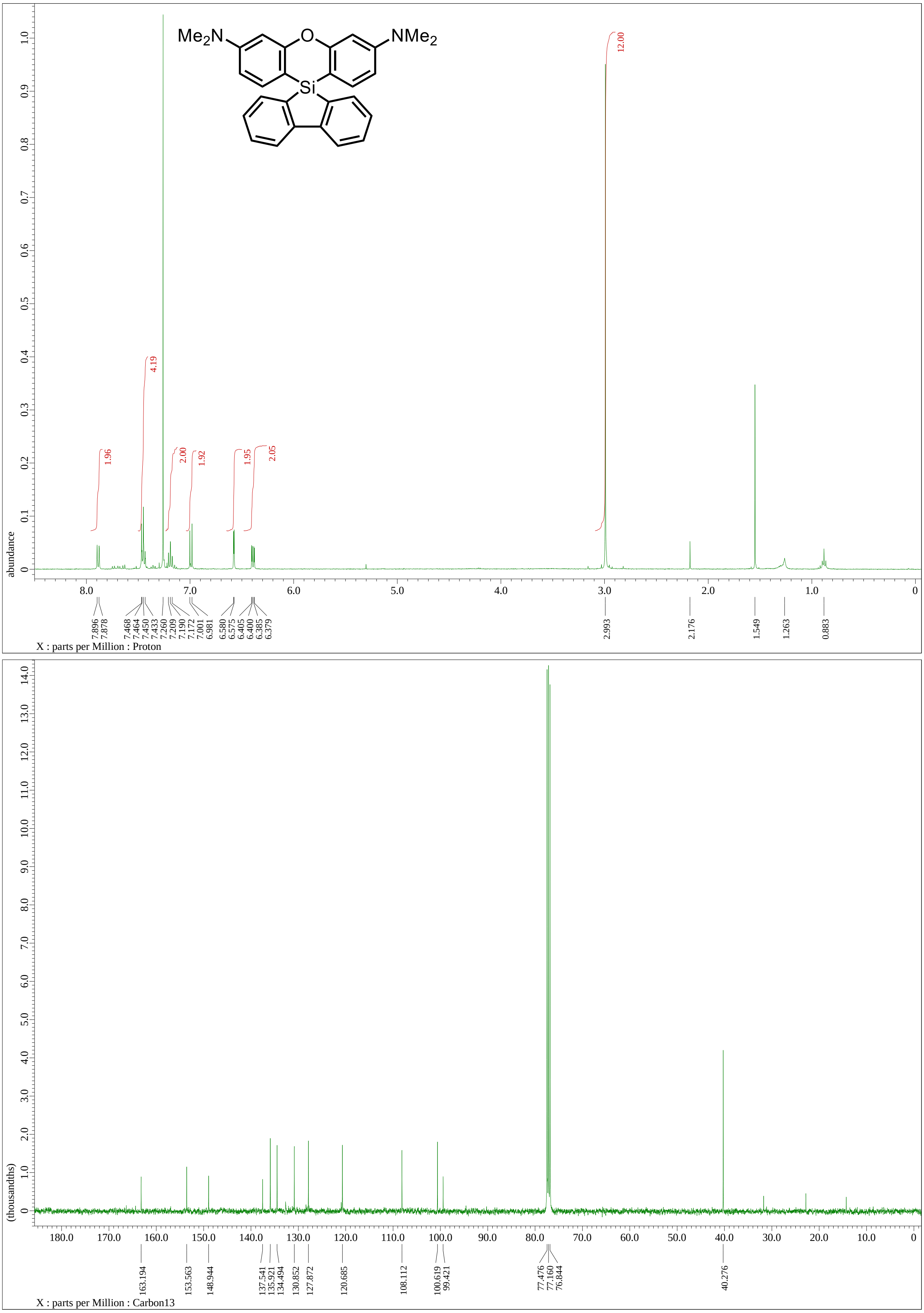

Figure S14: ^{1}H NMR (top) and ^{13}C NMR (bottom) of **3f**.

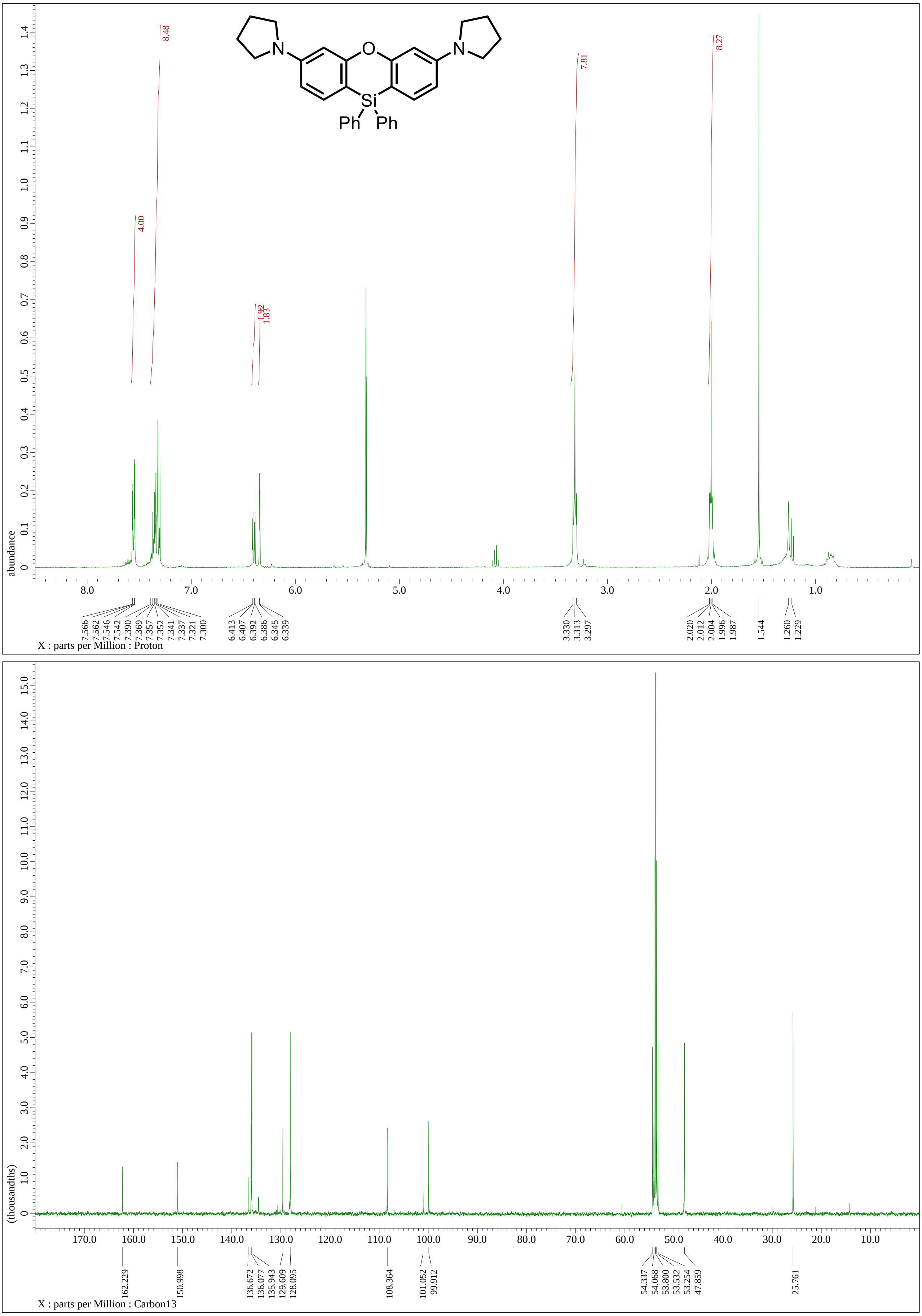

Figure S15: ^{1}H NMR (top) and ^{13}C NMR (bottom) of **3g**.

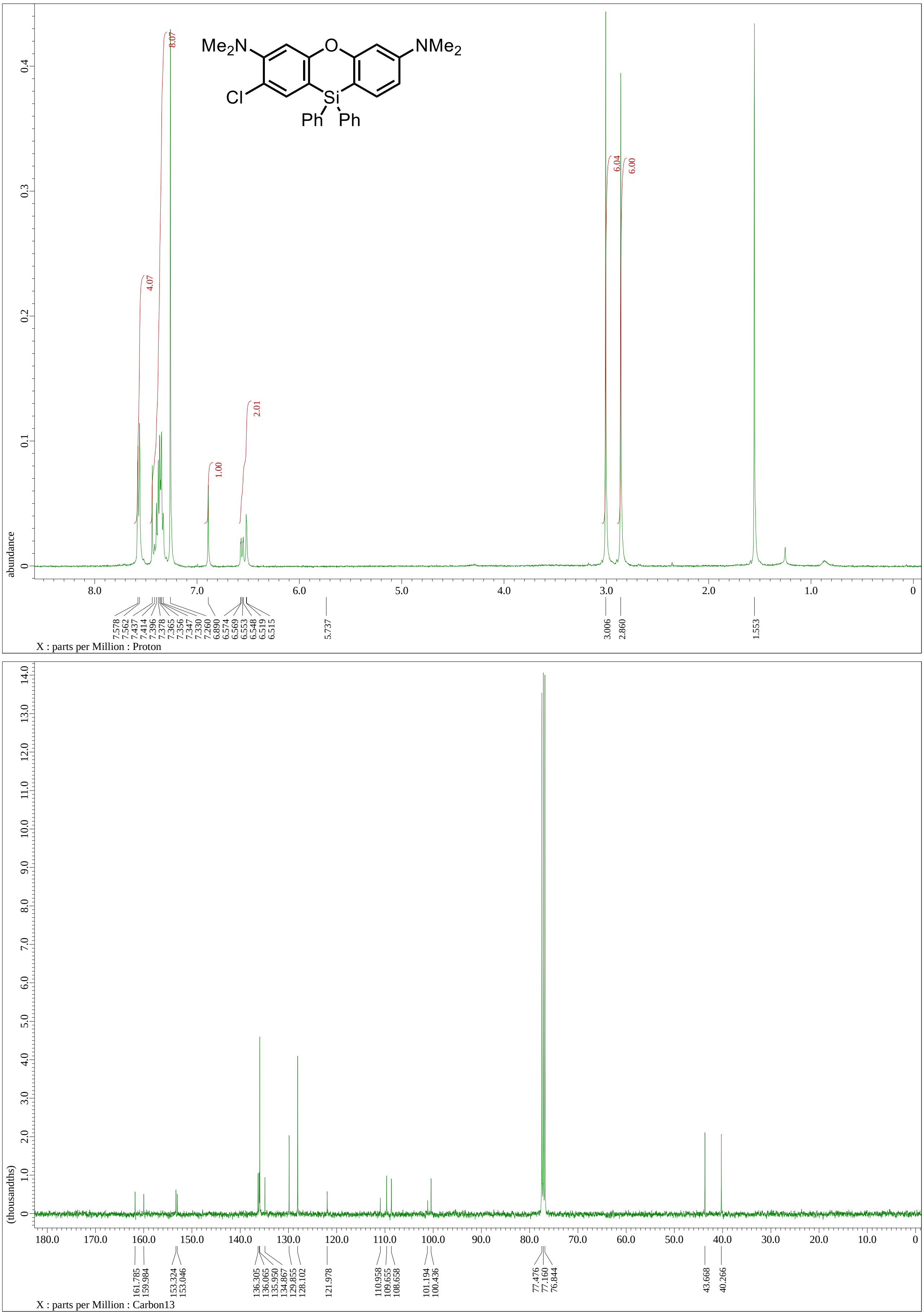

Figure S16: ^{1}H NMR (top) and ^{13}C NMR (bottom) of **3h**.

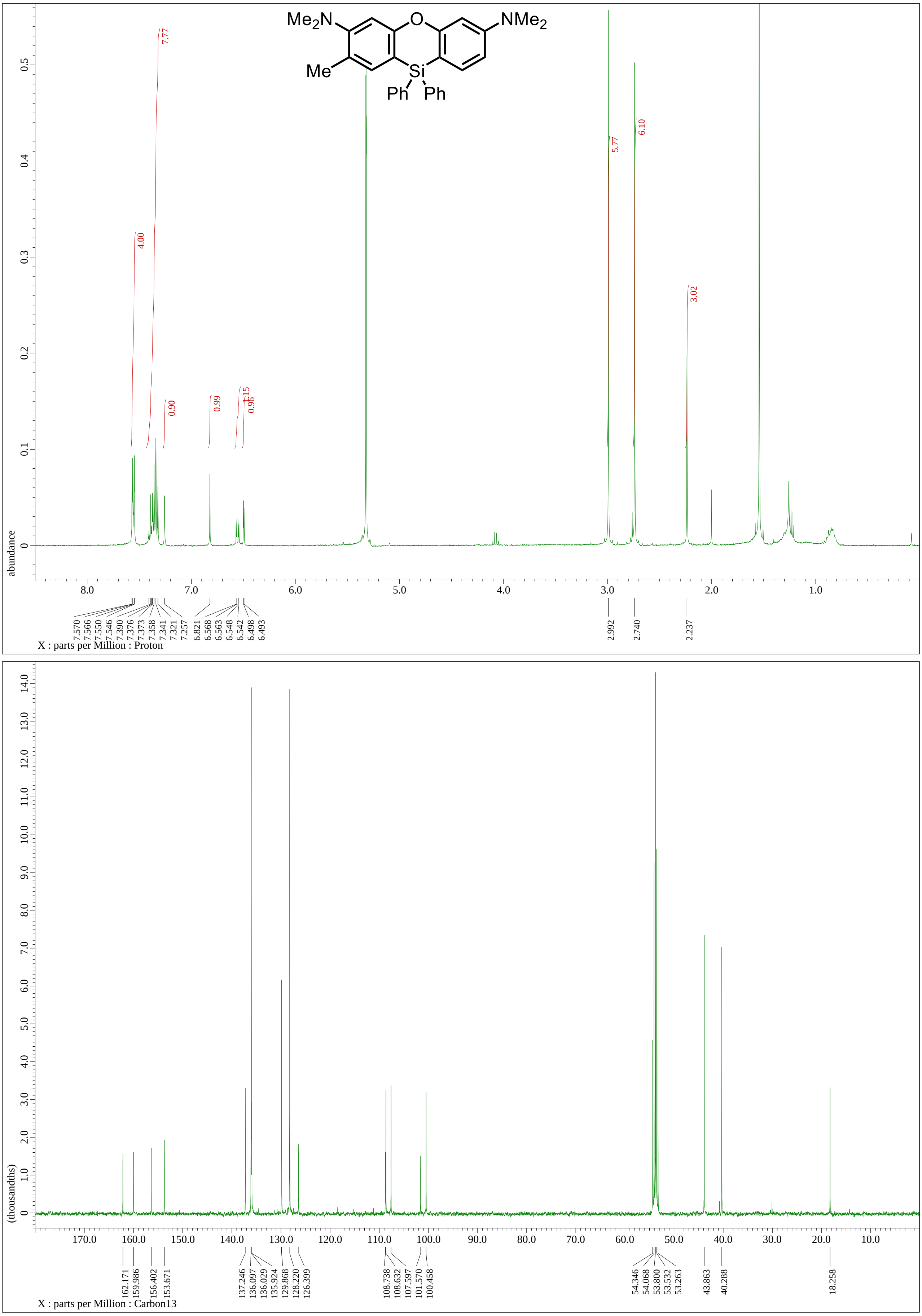

Figure S17: ^{1}H NMR (top) and ^{13}C NMR (bottom) of **3i**.

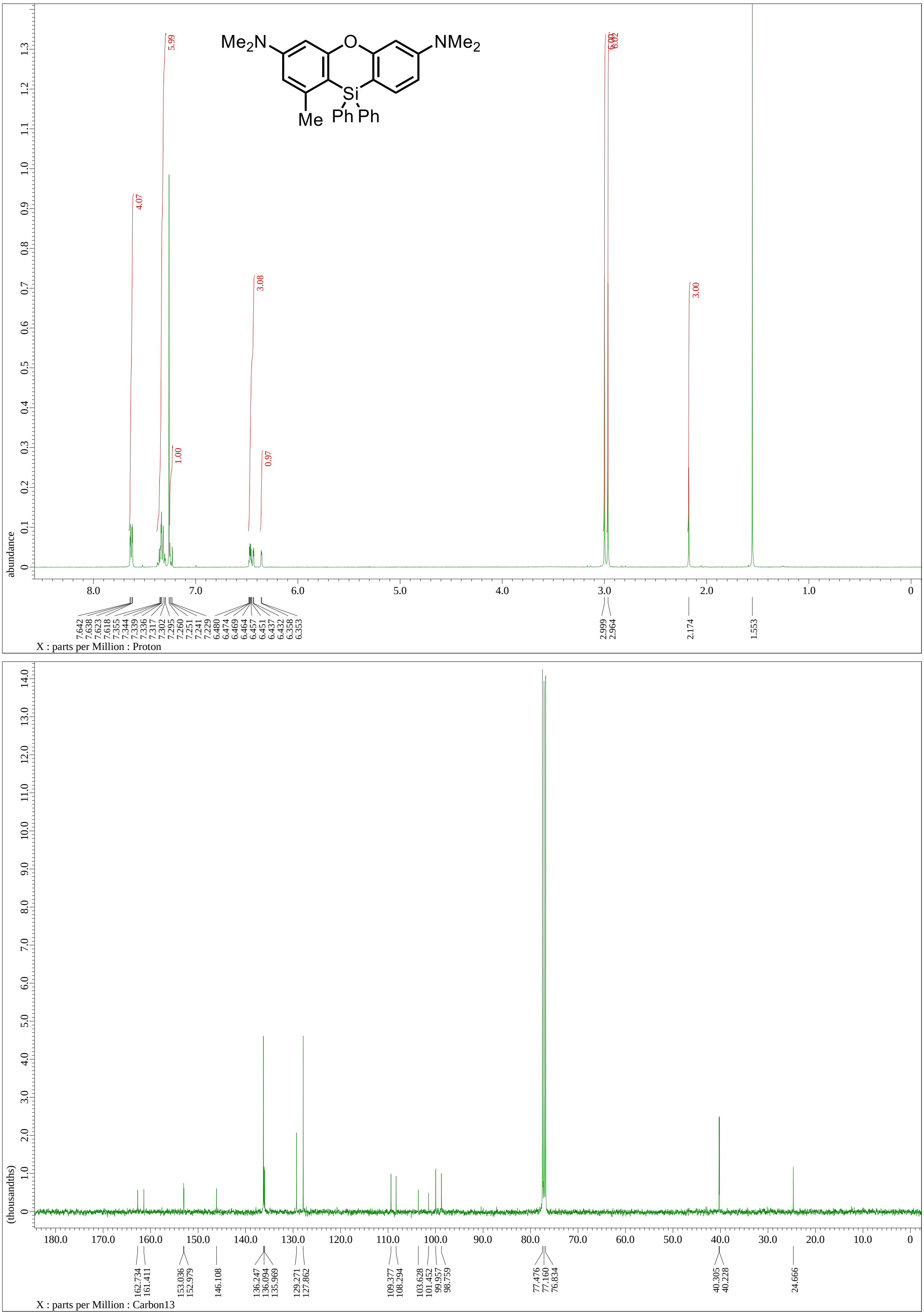

Figure S18: ^{1}H NMR (top) and ^{13}C NMR (bottom) of **3j**.

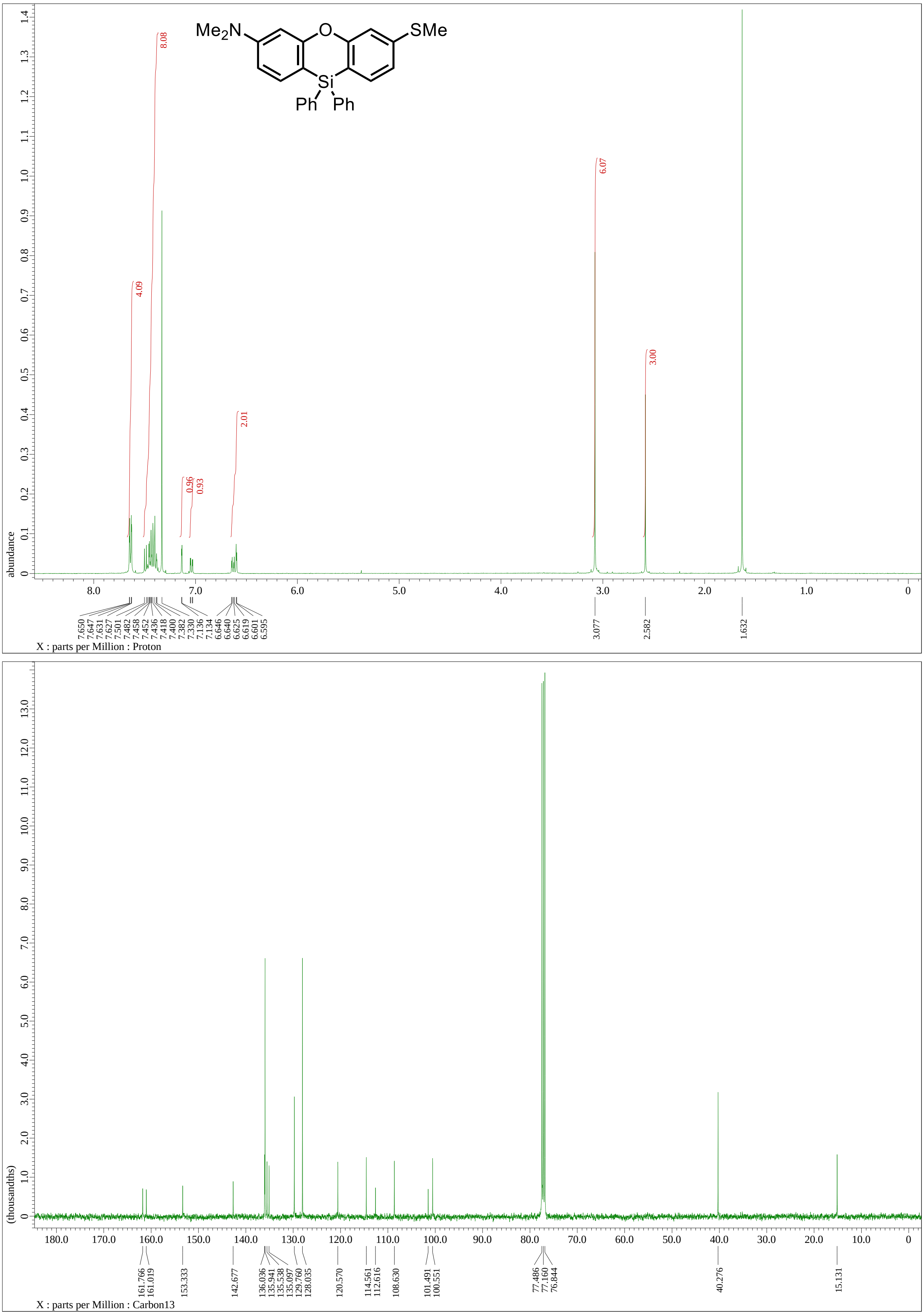

Figure S19: ^{1}H NMR (top) and ^{13}C NMR (bottom) of **3k**.

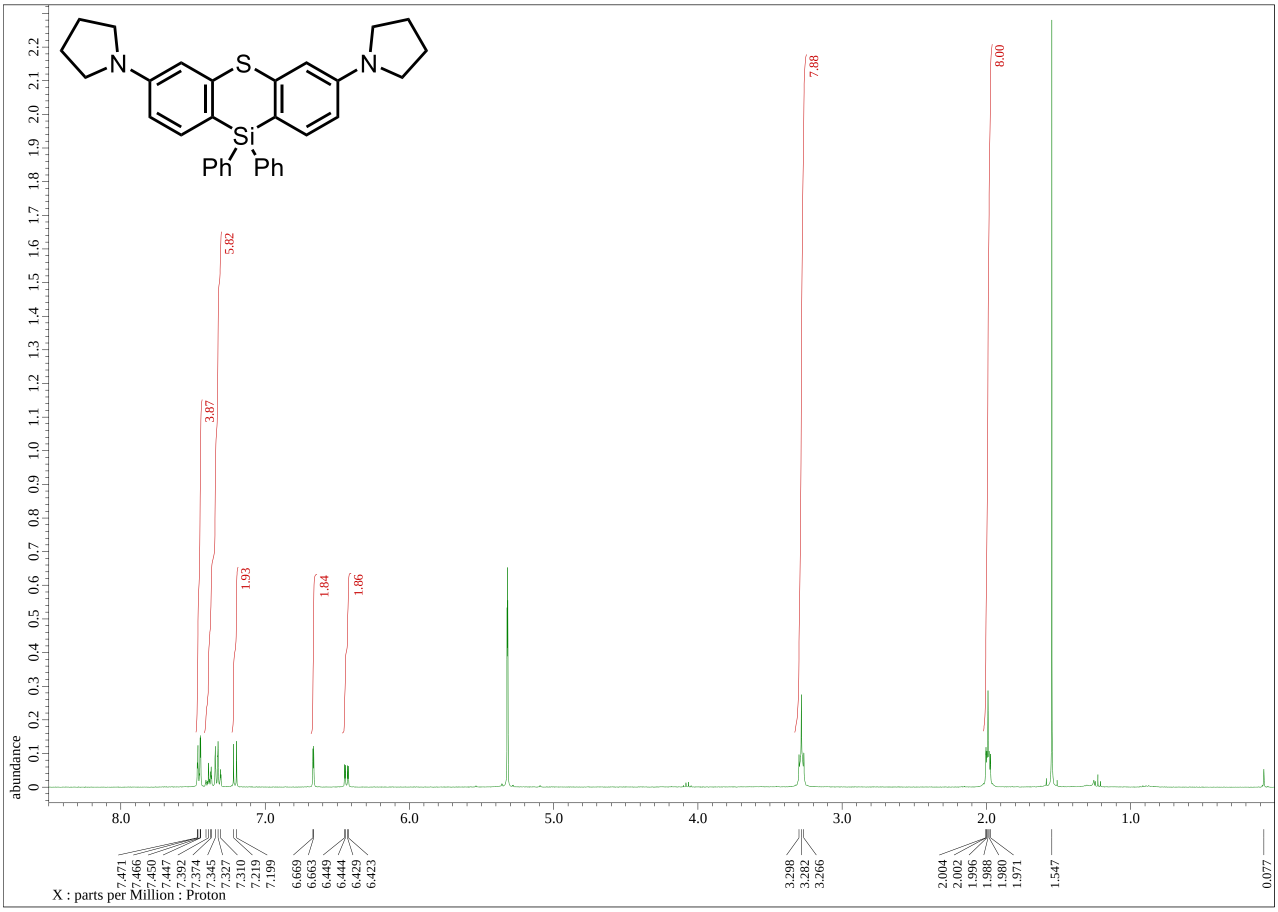

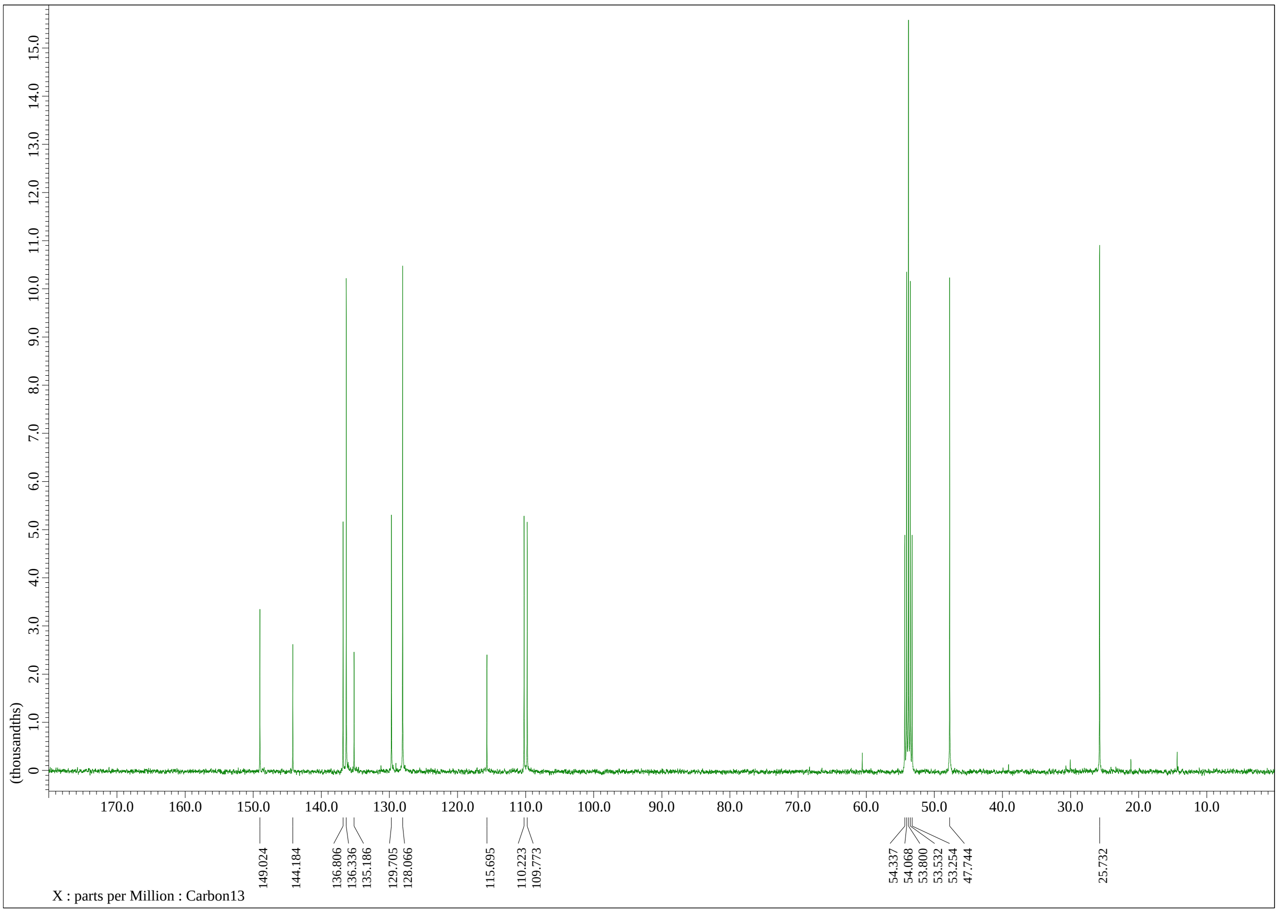

Figure S20: ^{1}H NMR (top) and ^{13}C NMR (bottom) of **3l**.

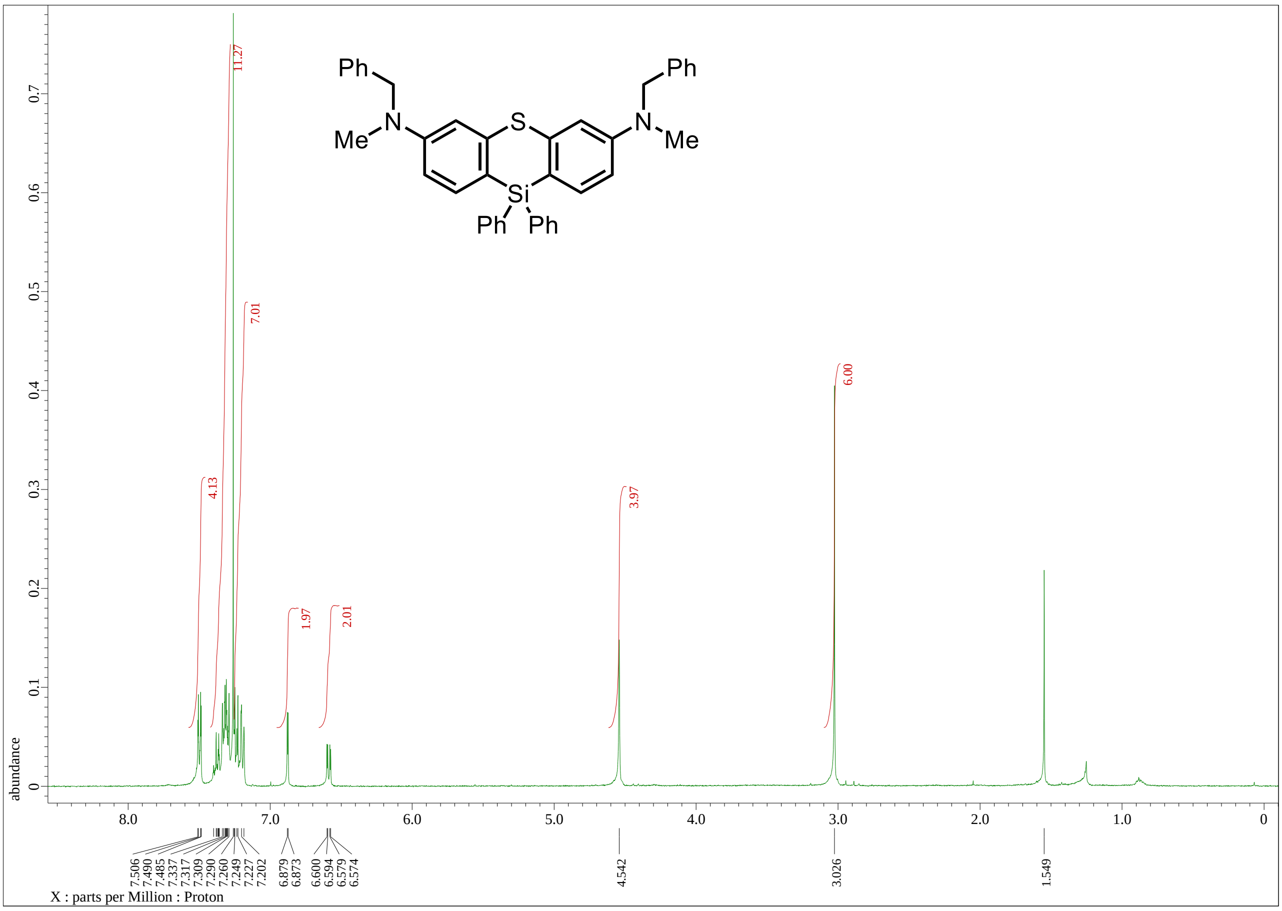

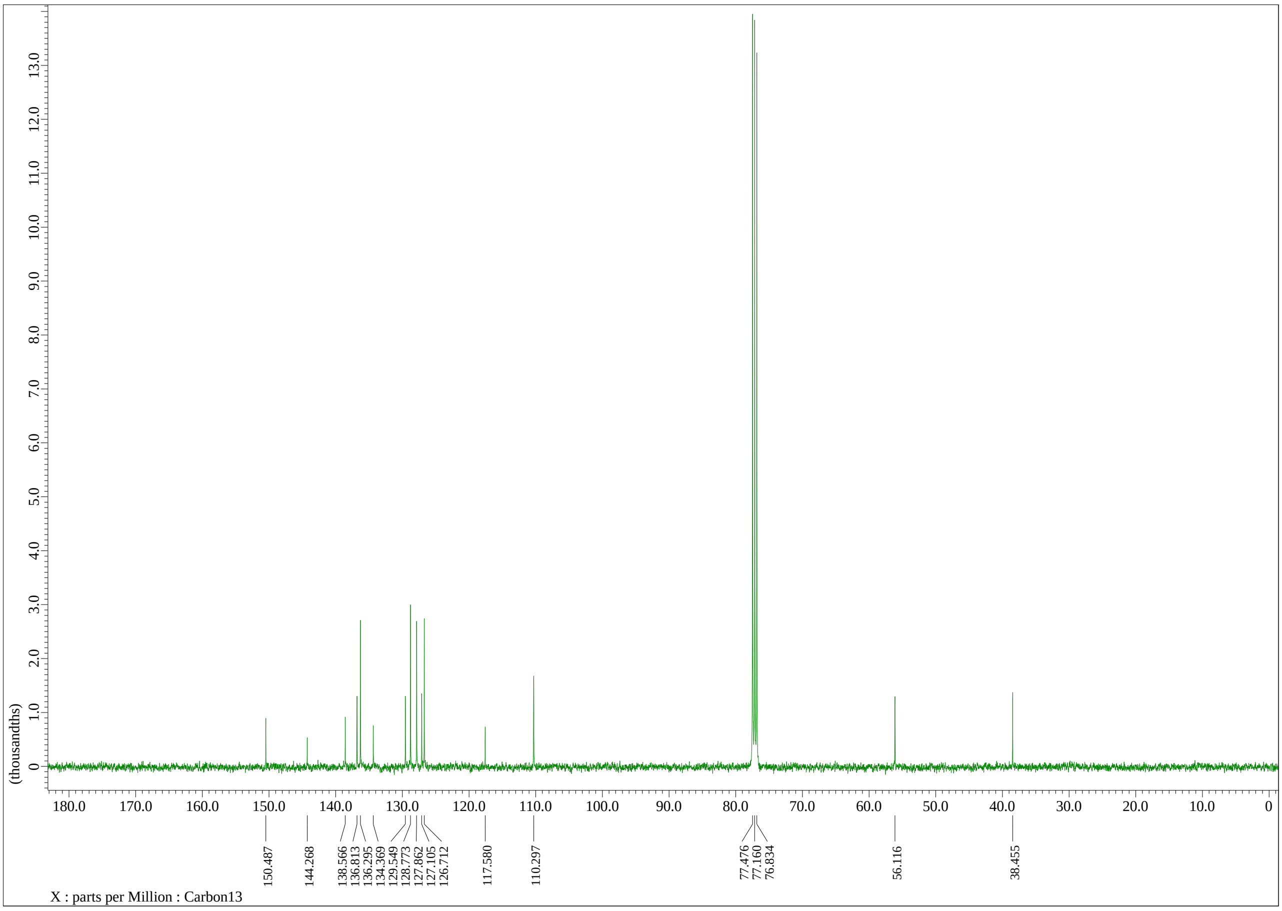

Figure S21: ^{1}H NMR (top) and ^{13}C NMR (bottom) of **3m**.

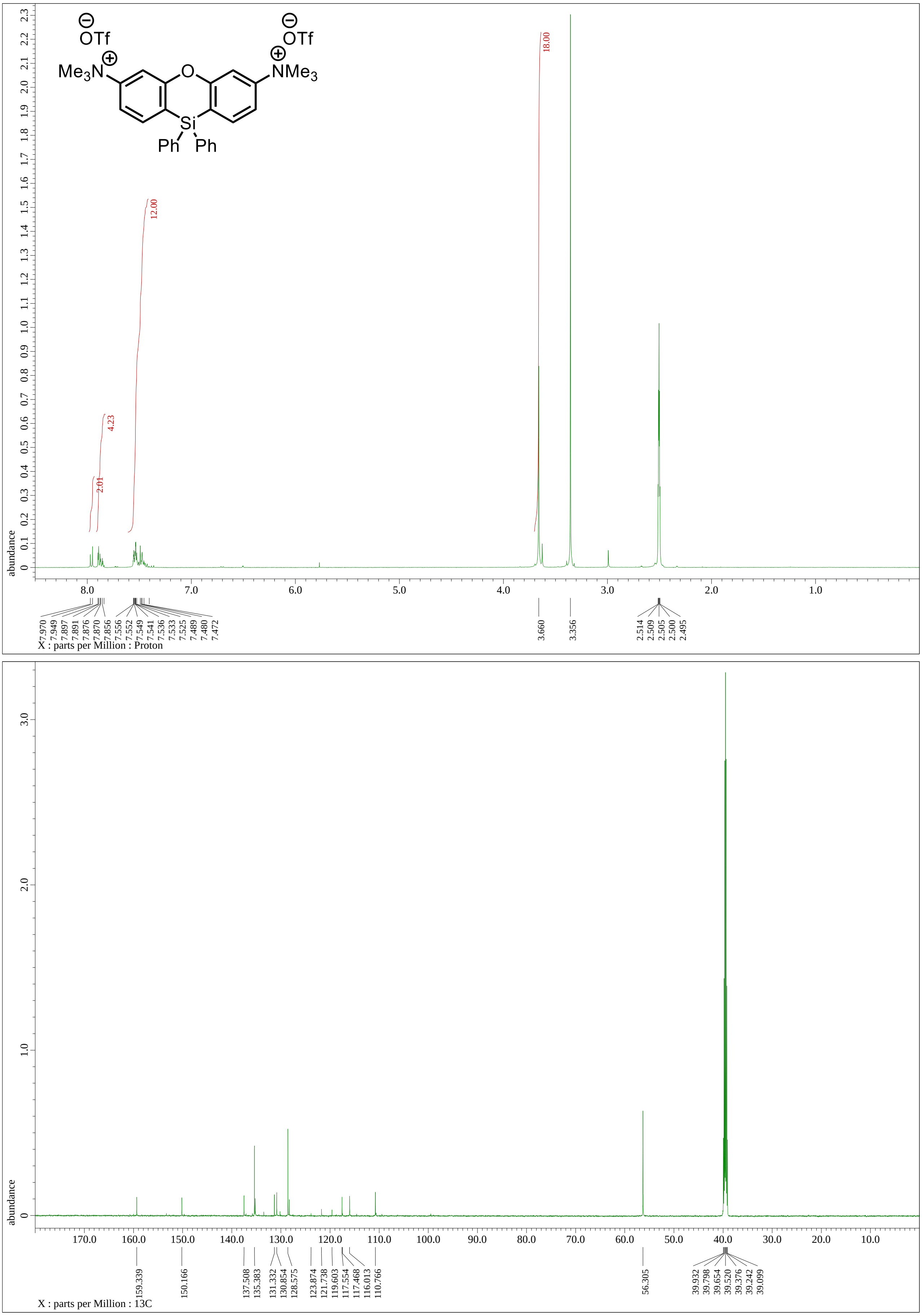

Figure S22: ^{1}H NMR (top) and ^{13}C NMR (bottom) of **4**.

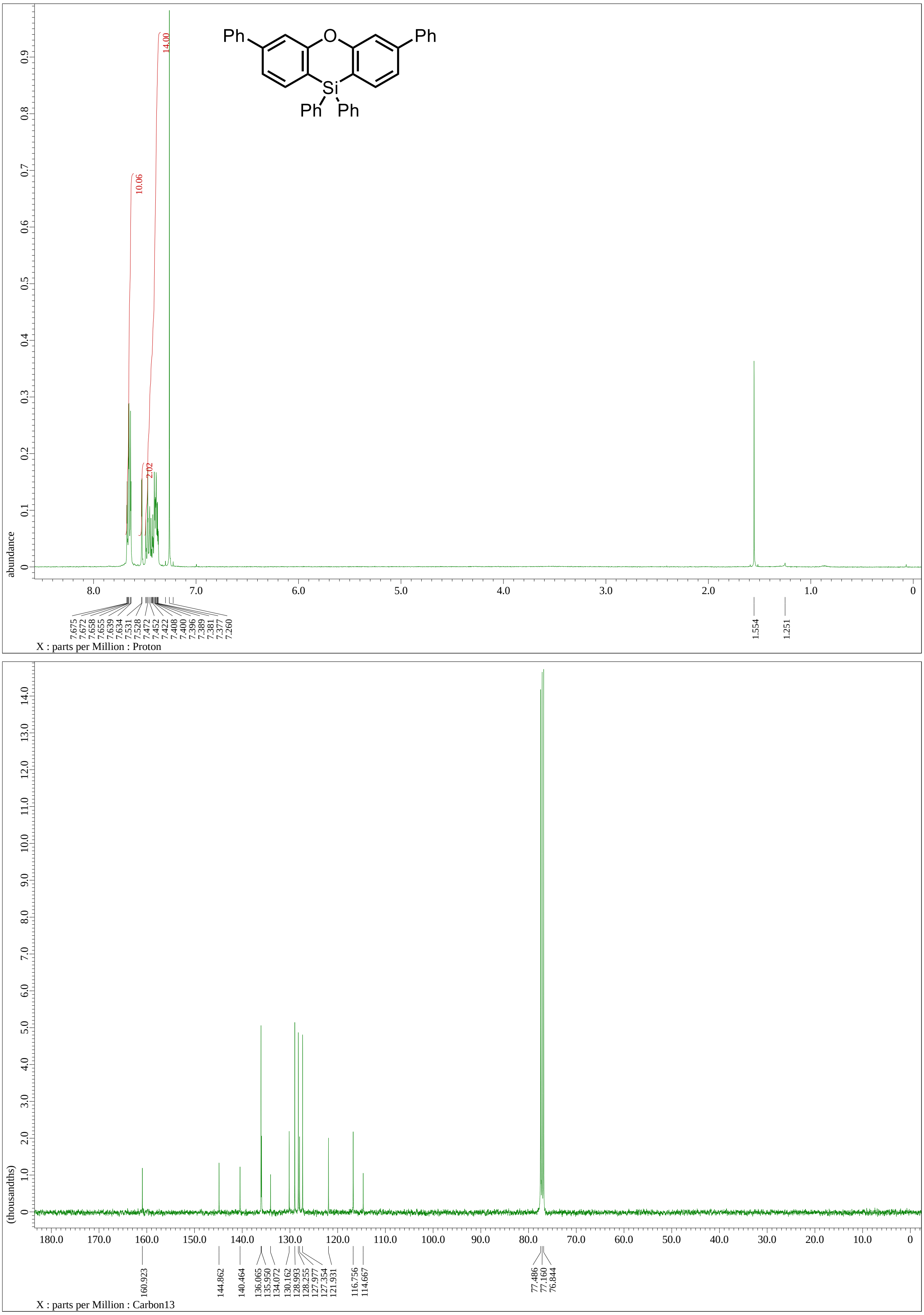

Figure S23: ^{1}H NMR (top) and ^{13}C NMR (bottom) of **5**.

6. References

[1] Yang, X.; Wang, C. *Angew. Chem. Int. Ed.* **2018**, *57*, 923–928.

[2] Corey, J. C.; John, C. S.; Ohmsted, M. C.; Chang, L. S. *J. Organomet. Chem.* **1986**, *304*, 93–105.

[3] Shen, H.; Zhang, X.; Liu, Q.; Pan, J.; Hu, W.; Xiong, Y.; Zhu, X. *Tetrahedron Lett.* **2015**, *56*, 5628–5631.

[4] Pastierik, T.; Šebej, P.; Medalová, J.; Štacko, P.; Klán, P. *J. Org. Chem.* **2014**, *79*, 3374–3382.

[5] Fischer, C.; Sparr, C. *Angew. Chem. Int. Ed.* **2018**, *57*, 2436–2440.

[6] Bin, X.; Mao-Lin, Li.; Xiao-Dong, Z.; Shou-Fei, Z.; Qi-Lin, Z. *J. Am. Chem. Soc.* **2015**, *137*, 8700−8703.

[7] Dong, Y.; Takata, Y.; Yoshigoe, Y.; Sekine, K.; Kuninobu, Y. *Chem. Commun.* **2019**, *55*, 13303–13306.

[8] Wolfe, J. P.; Buchwald, D. L. *J. Org. Chem.* **2000**, *65*, 1144−1157.

[9] Wu, X. M.; Hu W. Y. *Chin. Chem. Lett.* **2012**, *23*, 391−394.

[10] SIR2008: Burla, M. C.; Caliandro, R.; Camalli, M.; Carrozzini, B.; Cascarano, G. L.; De Caro, L.; Giacovazzo, C.; Polidori, G.; Siliqi, D.; Spagna, R. *J. Appl. Crystallogr.* **2007**, *40*, 609−613.

[11] DIRDIF99: Beurskens, P. T.; Admiraal, G.; Beurskens, G.; Bosman, W. P.; de Gelder, R.; Israel, R.; Smits, J. M. M.; The DIRDIF-99 program system; Technical Report of the Crystallography Laboratory; University of Nijmegen, Nijmegen, The Netherlands, **1999**.

[12] Cromer, D. T.; Waber, J. T. International Tables for X-ray Crystallography; Kynoch Press: Birmingham, U.K., **1974**, Vol. 4.

[13] Olex2 program package: Dolomanov, O. V.; Bourhis, L. J.; Gildea, R. J.; Howard, J. A. K.; Puschmann, H. *J. Appl. Cryst.* **2009**, *42*, 339−341.

[14] SHELX97: Sheldrick, G. M. *Acta Cryst.* **2008**, *A64*, 112.
